# EPLIN, a prospective oncogenic molecule with contribution to growth, migration and drug resistance in pancreatic cancer

**DOI:** 10.1038/s41598-024-81485-w

**Published:** 2024-12-28

**Authors:** Jianyuan Zeng, Cai Wang, Fiona Ruge, Edison Ke Ji, Tracey A. Martin, Andrew J. Sanders, Shuqin Jia, Chunyi Hao, Wen G. Jiang

**Affiliations:** 1 School of Medicine, Cardiff University, Henry Wellcome Building, Cardiff, CF14 4XN UK; 2 Gastrointestinal Cancer Centre, Peking University Cancer Hospital, Peking University, Fucheng Road, Haidian District, Beijing, China; 3 School of Education and Science, University of Gloucestershire, Francis Close Hall, Swindon Road, Cheltenham, GL50 4AZ UK

**Keywords:** EPLIN, Migration, Pancreatic cancer, PIK3s, MAPK, Chemotherapeutic resistance, Pancreatic cancer, Metastasis

## Abstract

Most pancreatic cancer patients are diagnosed at advanced stages, with poor survival rates and drug resistance making pancreatic cancer one of the highest causes of cancer death in the UK. Understanding the underlying mechanism behind its carcinogenesis, metastasis and drug resistance has become an essential task for researchers. We have discovered that a well-established tumour suppressor, EPLIN, has an oncogenic rather than suppressive role in pancreatic cancer. Notably, upregulation of EPLIN was observed in pancreatic cancer samples compared to normal samples at RNA and protein levels. Moreover, the presence of EPLIN resulted in poor clinical outcomes in patients. We also report that inhibition of EPLIN led to reduced cellular growth and migration in pancreatic cancer cells. EPLIN regulates expression and phosphorylation levels of several key players in MAPK and PIK3CA-AKT signalling pathways, as well as key contributors of EMT. Furthermore, EPLIN mediates the inhibitory ability PIK3 kinases, MEK and ERK inhibitors have on cell migration. EPLIN was also found to have an impact on pancreatic cancer cells response to chemotherapeutic and EGFR/HER2 targeted therapeutic agents, namely gemcitabine, fluorouracil (5FU) and neratinib (Nerlynx).

**Supplementary Information:**

The online version contains supplementary material available at 10.1038/s41598-024-81485-w.

## Introduction

Pancreatic cancer refers to malignant tumours occurring in the pancreas, over 90% of which are pancreatic ductal adenocarcinoma (PDAC)^1^. Pancreatic cancer is rated as the 5th leading cause of cancer death in the UK^2^. Unfortunately, Over 60% of pancreatic cancer patients are diagnosed at the most aggressive stage (Stage IV)^3^. Furthermore, the 5-year survival rate of pancreatic cancer patients is less than 5% and has not been improved since the 1970s^2^. The unsettling incidence rates and prognosis of pancreatic cancer urges researchers to investigate novel therapeutic strategies thus, to improve the clinical outcomes. The genetic alterations in pancreatic cancer often include the mutation of an oncogene, *KRAS*,* with* deregulation/inactivation of several tumour suppressors, namely *BRCA1/2*,* p53*,* CDKN2A* and *SMAD4*^4^. Activation of relative signalling events, due to such genetic alterations such as TCF-β, EGFR/KRAS and WNT, leads to the development of the malignant tumour (such as proliferation and epithelial mesenchymal transition (EMT)) and metastasis^5^. The predominant treatment strategy for pancreatic cancer is surgical radical resection^6^. Neoadjuvant chemotherapy is one of the essential strategies to promote patient clinical outcomes, in which gemcitabine, paclitaxel and modified FOLFIRINOX (leucovorin, 5-fluorouracil, irinotecan, and oxaliplatin) are utilised as first-line treatment^7,8^. However, despite profound improvements due to such therapeutic agents, chemotherapeutic resistance against drugs such as gemcitabine directly challenges curative effects^9^. In recent years, novel therapies such as targeted therapy and immunotherapy have also become part of the therapeutic regime eme^10^. Notably, inhibitors/antagonists against oncogenes such as tropomyosin receptor kinase (TRK), Ki-ras2 Kirsten rat sarcoma viral oncogene homolog (KARS)^10^, epidermal growth factor receptor (EGFR)/ epidermal growth factor receptor 2 (Her2)^11^ have been studied by researchers and shed light on potential novel therapeutic strategies for pancreatic cancer.

Epithelial protein lost in neoplasm (EPLIN) is encoded by the *LIMA1* gene and it was initially reported to be downregulated in oral cancer^12,13^. EPLIN bundles actin filaments via actin binding sites located at the LIM domain and stabilizes actin dynamics by inhibiting actin polymerization via the Arp2/3 complex^14^. EPLIN also directly links to the cadherin-catenin complex through α-catenin, and therefore regulates adherens junctions (AJ)^15^. Disorganisation of AJ and disassembly of the cadherin-catenin complex can be induced by inhibition or phosphorylation of EPLIN through its upstream regulators (e.g. extracellular signal- regulated kinase (ERK))^16^. Furthermore, EPLIN has been described as a tumour suppressor, as the downregulation of EPLIN and induced deregulation of downstream participants results in promotion of cellular functions and the EMT process in multiple cancer types^15–23^. For instance, the loss or mutation of p53 leads to inhibition of EPLIN and results in promotion of invasion of lung cancer cells^21^. Inhibition of EPLIN results in upregulation of ZEB1 and β-catenin in prostate cancer^16,18^, as well as upregulation of Slug in melanoma cells^22^. Additionally, clinical studies revealed that downregulation of EPLIN in tumour samples resulted in poor clinical outcomes in breast cancer^24^, prostate cancer^20,25^, lung cancer^26^, ovarian cancer^27^ and colorectal cancer^23^. EPLIN was also implied to have an impact on chemotherapeutic resistance in gastric cancer^28^ and colorectal cancer^23^.

The current study explored the expression of EPLIN in clinical pancreatic cancer tissues and investigated the functions of EPLIN in pancreatic cancer cells. Here, we report that instead of being tumour suppressive, EPLIN plays a putative oncogenic role in pancreatic cancer. Upregulated EPLIN expression level was observed in pancreatic cancer and such upregulation would lead to poor clinical outcomes. Moreover, EPLIN regulates cellular growth and migration positively in pancreatic cancer cells. By employing inhibitors for phosphoinositide 3-kinases (PIK3s), ERK and mitogen-activated protein kinase kinase (MEK), knocking down EPLIN in pancreatic cancer cell lines inhibited inhibitors’ effect on cellular migration. Knocking down EPLIN downregulates key components in MAPK and PIK3 signalling events at protein levels. Regulatory relationship between EPLIN and several key regulators of EMT, namely SNAIL, SLUG and ZEB1 in pancreatic cancer was also demonstrated. Furthermore, EPLIN has an impact on cellular response to chemotherapeutic and EGFR/Her2 targeted therapeutic agents. Thus, in the current study, we suggest that EPLIN acts as a putative oncogene in pancreatic cancer and a upstream regulator of MAPK and PIK3CA-AKT signalling events.

## Materials and methods

### Collection of the pancreatic cancer clinical cohort

A pancreatic cancer cohort of one hundred and ninety nine PDAC patients: Following the approval of the Ethics Research Committee of Peking University Cancer Hospital (Ethics approval number: 2006021), tumour samples and adjacent normal tissues were harvested after surgery and stored in liquid nitrogen until required. The procedure was carried out fully in accordance with relevant guidelines and regulations and was performed in accordance with the Helsinki declarations. The current cohort has a median follow-up period of 12 months.

### Cell culture

Low passage (< 15) pancreatic cancer cell lines, namely MIAPaCa2 and PANC1 (ATCC, Rockville, MD, USA) were cultured for the current study. Cell lines were cultured in Dulbecco’s Modified Eagle’s medium (DMEM) which was supplemented with 10% heat inactivated foetal calf serum (FCS) (Sigma-Aldrich, Poole, Dorset, UK) and 1% of a 100X antibiotic mixture including penicillin, streptomycin and amphotericin B (Sigma-Aldrich, Poole, Dorset, UK). Cell culture was carried out at 37 °C with 95% humidity and 5% CO_2_.

### Generation of EPLIN knockdown cell models with lentiviral transfection

Manipulation of EPLIN expression in pancreatic cancer cell lines was achieved by performing lentiviral transfection with EPLIN shRNA lentiviral particles (sc-60593-V. Insight Biotechnology Limited, Middlesex, UK). Briefly, cells were seeded into a 24-well plate and were allowed to reach around 50% confluence. The shRNA lentiviral particles were added following the manufacturer’s instructions. To enhance the efficiency of transfection, 1: 100 of 8 µg/ml polybrene was also added. Transfected cells were selected by 2 µg/mL puromycin and were maintained in culture medium containing 0.2 µg/mL puromycin.

### Reverse transcription

RNA from patients’ tissue samples and pancreatic cancer cell models was isolated with TRI Reagent (Sigma-Aldrich, Poole, Dorset, UK) following with the manufacturer’s guidelines. RNA samples were then standardised to 500ng/µl for reverse transcription (RT). RT was carried out using the GoScript™ Reverse Transcription System Kit (Promega, Southampton, UK) in a Slimpliamp thermocycler (Fisher Scientific UK Leicestershire, UK). Once completed, cDNA samples were stored at -20 °C until use.

### Conventional polymerase chain reaction (PCR) and real-time PCR

cDNA samples were used to examine the transcript expression level of EPLIN by performing conventional PCR and qPCR. For conventional PCR, 1 µl cDNA sample was mixed with 1 µl forward primers (EPLIN: TCAAACTAAGATTCTCCGGG. EPLINβ: CATTTAATAGACGGCAATGGA. GAPDH: GGCTGCTTTTAACTCTGGTA), 1 µl reverse primers (EPLIN: CAATAGGGGCATCTTCTACC. EPLINβ: CCGGAGAATCTTAGTTTGAGT. GAPDH: GACTGTGGTCATGAGTCCTT) (Sigma-Aldrich, Poole, Dorset, UK), 5 µl PCR water and 8 µl PCR GoTaq Green master mix (Promega, Southampton, UK) for each reaction. The solution was then placed in a Slimpliamp thermocycler (Fisher Scientific UK, Leicestershire, UK) for a 32 cycle PCR. PCR products were then separated using for Agarose gel electrophoresis. The gel was transferred to a Syngene U: Genius 3 Fluorescence UV Transilluminator (Synoptics Ltd., Cambridge, UK) for visualisation.

EPLIN transcript expression level was also tested by Real-time PCR (qPCR) using the Amplifilour Uniprimer™ Universal system (Intergen company, New York, USA). In brief, each reaction contained FAST2x qPCR Master Mix (PrimerDesign, Southampton, UK), forward primers (EPLIN: AAGCAAAAATGAAAACGAAG. GAPDH: AAGGTCATCCATGACAACTT. PIK3CA: GTAGCCCAGATGTATTGCTT. EGFR: TCTTCGGGGAGCAGCGAT. HER2: CCTCCTCGCCCTCTTG. HER3: CCCCACACCAAGTATCAGTA. HER4: CTGCTGAGTTTTCAAGGATG. ERK1: TCTAAAGCCCTCCAACCT. ERK2: CCAACCTCTCGTACATCG. AKT1: CTACTACGCCATGAAGATCC. SLUG: TGGACACACATACAGTGATT. ZEB1: GTGTGGAAAAGCTTTCAAAT. SNAIL: CGCTCTTTCCTCGTCAG), reverse primer with z sequence (1/10) (EPLIN: ACTGAACCTGACCGTACAGACACCCACCTTAGCAATAG. GAPDH: ACTGAACCTGACCGTACAGCCATCCACAGTCTTCTG. PIK3CA: ACTGAACCTGACCGTACACAAAACCTCGAACCATAGGA. EGFR: ACTGAACCTGACCGTACACGTGAGCTTGTTACTGGTGC. HER2: ACTGAACCTGACCGTACACATGTCCAGGTGGGTCT. HER3: CTGAACCTGACCGTACAACACAGGATGTTTGATCCAC. HER4: CTGAACCTGACCGTACAAACTTGCTGTCATTTGGACT. ERK1: ACTGAACCTGACCGTACACCACATACTCCGTCAGGA. ERK2: ACTGAACCTGACCGTACAGGGGCTGATTTTCTTGAT. AKT1: ACTGAACCTGACCGTACAGGTCTGGAAAGAGTACTTCAG. SLUG: ACTGAACCTGACCGTACAGGATCTCTGGTTGTGGTATG. SNAIL: ACTGAACCTGACCGTACAAAACTCTGCATTAGAGTCCTGC. ZEB1: ACTGAACCTGACCGTACAGTGAGCTATAGGAGCCAGAA.) (Sigma-Aldrich, Poole, Dorset, UK), Uniprimer and cDNA mixture. Reaction mixture was used to run qPCR in a Step One Plus Real Time PCR System (Fisher Scientific UK Leicestershire, UK). A set of serial-diluted standard samples also underwent qPCR along with the test samples, in order to calculate relative transcript levels.

### Preparation of protein samples and western blotting

Pancreatic cancer cells were detached and collected, in phosphate-buffered saline (PBS) (Sigma-Aldrich Co, Poole, Dorset, UK), from tissue culture flasks using a rubber scraper and were centrifuged at 2,000 rpm for 7 min at room temperature. Supernatant was aspirated before radioimmunoprecipitation assay (RIPA) lysis buffer added and utilised to resuspend the cell pellet. Samples were put on a rotating wheel at 4 °C overnight before centrifugation at 13,000 rpm for 15 min at 4 °C. The supernatant was collected and quantified for western blotting.

Protein samples and BLUeye Prestained Protein Ladder (Geneflow Ltd., Litchfield, UK) were loaded into a two-layer gel, a layer of 5% stacking gel and a layer of 8% resolving gel, before performing sodium dodecyl sulfate-polyacrylamide gel electrophoresis (SDS-PAGE) at 120 V, 50 W and 50 mA to produce sufficient separation of proteins. Semi-dry protein transfer was performed to transfer protein from the gel to a polyvinylidene fluoride (PVDF) membrane (Merck Millipore, Hertfordshire, UK), in a semi-dry transfer apparatus at 15 V, 500 mA, 20 W for 50 min. The membrane was then blocked using 10% milk solution (10% milk in Tris buffered saline (TBS) and 0.1% tween-20 (Sigma-Aldrich Co, Poole, Dorset, UK)). The membrane was then incubated with 1:500 EPLIN (mouse monoclonal, sc-136399) or 1: 1000 GAPDH (mouse monoclonal, sc-32233) or 1:250 KRAS (mouse monoclonal, sc-30) or 1:250 EGFR (mouse monoclonal, sc-71034) or 1:250 HER2/NEU (mouse monoclonal, sc-33684) or 1:250 ERK1/2 (mouse monoclonal, sc-514302) or 1:250 p-ERK1/2 (mouse monoclonal, sc-7383) or 1:250 AKT1 (mouse monoclonal, sc-5298) or MEK2 (mouse monoclonal, sc-13159) (Insight Biotechnology Limited, Middlesex, UK) antibody overnight at 4 °C. Membrane washing was performed with 3% milk solution at room temperature for 15 min, three times. The membrane was incubated with a secondary antibody (Rabbit anti-mouse (whole molecule) IgG peroxidise conjugate, A5278, Sigma-Aldrich Co, Poole, Dorset, UK) for 1 h at room temperature before being washed with TBS-T (TBS with 0.2% tween-20) and TBS. The membrane was incubated with EZ-ECL solution (Geneflow Ltd., Litchfield, UK) in the dark before using a G-BOX (Syngene, Cambridge, UK) detection system to capture pictures of protein bands.

### Immunohistochemical (IHC) assay on the pancreatic cancer tissue microarray (TMA)

A pancreatic cancer TMA (PA2081a), purchased from US Biomax, Inc. (Derwood, MD, USA), was utilised for IHC staining and subsequent analysis of EPLIN expression levels. The TMA contained 192 samples from 96 patients. For the IHC staining, the TMA was mounted with a glass slide and fixed in acetone for 15 min. After drying at room temperature, the TMA was rehydrated and washed with PBS before 0.1% Saponin solution (in TBS) was used for permeabilization. The TMA was then blocked with 10% horse serum for an hour before incubating with EPLIN antibody (mouse monoclonal, sc-136399, Insight Biotechnology Limited, Middlesex, UK) for an hour. After washing, it was then incubated with secondary antibody for 30 min, before the staining was developed using a avidin-biotin complex (ABC) reagent in VECTASTAIN^®^ ABC Kit (Vector Laboratories, Inc., CA, USA) and 3,3' diaminobenzidine (DAB) substrate (5 mg/ml). Stringent washing was performed between each process. Gill’s haematoxylin (Vector Laboratories Inc, CA, USA) was then used to counterstain the TMA slide before rehydration. The staining of the TMA was assessed by two independent researchers as previously reported^29^.

### Thiazolyl blue tetrazolium bromide (MTT) based cellular growth assay

Pancreatic cancer cell models were assessed for cellular growth using a MTT based assay to investigate the implication of EPLIN. In brief, 3,000 cells from each of the MIAPaCa2 cell models and 5,000 cells from PANC1 models were seeded in two 96-well plates in 8 repeats. After incubating for 24 h in the incubator, 0.5 mg/ml MTT solution (Sigma- Aldrich Co, Poole, Dorset, UK) was added into each well containing cells in one of the plates. After further incubation for 4 h, medium was discarded and 100 µl DMSO (Sigma- Aldrich Co, Poole, Dorset, UK) was supplemented. Absorbance was then detected in an LT4500 plate reader (Wolf Laboratories, York, UK) at 540 nm. After 3 days, a second plate was processed to assess absorbance.

### Electric cell-substrate impedance sensing (ECIS) based cell migration assay

ECIS was carried out to assess migratory ability by creating wounds electrically. 30,000 to 40,000 cells were seeded into a 96-well ECIS W961E electrode array in 6 repeats. The plate was placed on the ECIS Zθ instrument (Applied Biophysics Ltd, Troy, New Jersey, USA) and incubated at 37 °C for 4–5 h, until a confluent monolayer was formed. ECIS created an electrical wound (2000 mA for 20 s) in each well via the electrode at the bottom of the plate and impendence was measured and recorded immediately. The measurement across 1,000 to 64,000 Hz was carried out by the ECIS system over 15 h. Data was analysed by the ECIS software.

### Wound scratching assay for cellular migration

A wound scratching assay was also performed to investigate migration ability. In brief, 300,000 to 400,000 cells from each model were seeded into a 96-well plate in duplicate and allowed to incubate at 37 °C with 5% CO_2_ for 24 h, to reach a confluent monolayer. Two vertical wounds were created by using a pipette tip. Each well was washed with PBS before supplementing with fresh medium. Wortmannin, LY294002, PD98059 and ravoxertinib (Bio-Techne Ltd., Abingdon, UK) were also supplemented in tested groups at 10nM, 500nM, 2µM and 60nM respectively. Following creation of the wounds, the plates were added to the EVOS systems which was programmed to capture images at specified locations on each of the wells over a range of time points, allowing monitoring of wound closure over time. ImageJ was used to analyse the wounded areas of each picture.

### Cytotoxicity assay

Cell models were used to perform cytotoxicity assays to assess the ability of cell to respond to therapeutic agents. Three thousand cells from MIAPaCa2 cell models or 5,000 cells from PANC1 cell models were seeded into a 96-well plate in triplicate. Chemotherapeutic and EGFR/HER2 targeted therapeutic agents, namely gemcitabine, 5FU (Sigma-Aldrich, Poole, Dorset, UK) and neratinib were also seeded in the 96-well plate in triplicate, in serial dilution. After incubating for 72 h, 0.5 mg/ml MTT solution was supplemented into each well for a further 4-hour incubation at 37 °C with 5% CO_2_. Medium was aspirated and DMSO was added before absorbance was detected on an LT4500 plate reader at 540 nm.

### Statistics

Several statistical softwares were carried out in the current study for the statistical analysis. ImageJ (https://imagej.nih.gov/ij) was used for semi-quantification of the PCR and WB pictures as well as the analysis of wound scratching assays. GraphPad (Prism 10) (GraphPad Software, San Diego, CA, USA) was carried out to perform two-tailed T-test and and Chi-square (χ^2^) test, Minitab (Minitab Ltd. Coventry, UK) and SPSS version 26 (IBM, Armonk, New York, USA) were used for analysing the transcript level of EPLIN in comparison of pathological information and performing Kaplan-Meier survival curve.

## Results

### EPLIN expression is upregulated in pancreatic cancer

A pancreatic cancer clinical cohort that contained 146 normal tissues and 199 tumour tissues was used to assess the expression profile of EPLIN in comparison to patient pathological information (Table 1). As Table 1 shows, no significance is noted in the expression level between normal tissues and tumour tissues. However, the transcript levels of EPLIN in moderate and low differentiated samples were higher than those which were highly and moderately differentiated (*p* = 0.025). Similarly, tissues in TNM2 had a higher expression of EPLIN than tissues in TNM1 (*p* = 0.031). A near statistical significance was also observed between tissues in T1/T2 and T3/T4. The expression of EPLIN in T3/T4 group was higher than in T1/T2 group (*p* = 0.064).

**Fig. 1:**
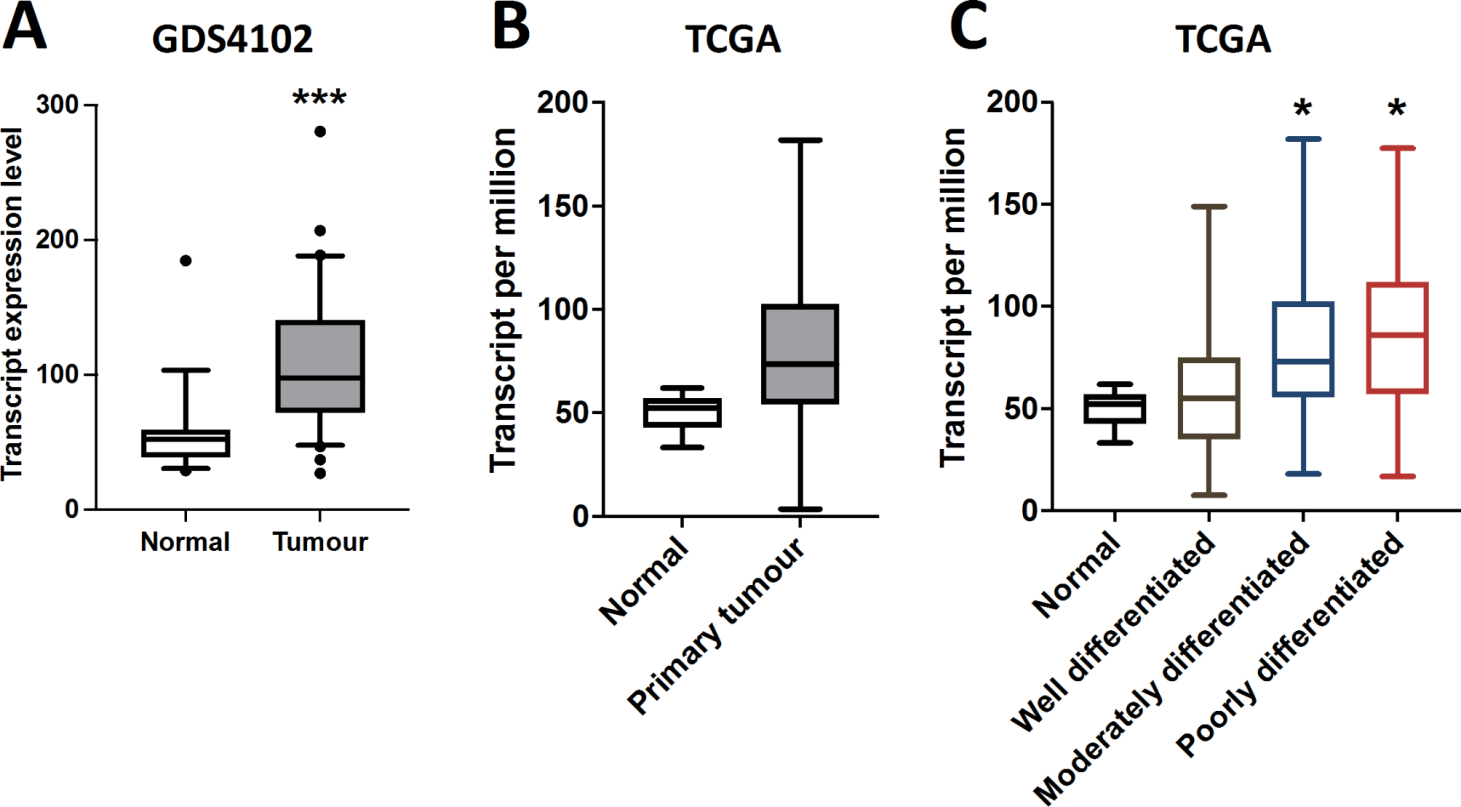
Transcript expression profile of EPLIN in pancreatic cancer public databases. (**A**) EPLIN transcript expression in pancreatic cancer GEO dataset (GDS4102). Normal samples: *n* = 16, median = 52.2, q1 = 38.9, q3 = 59.52; Tumour samples: *n* = 36, median = 97.64, q1 = 72.04, q3 = 140.8; *p* < 0.001. (**B**) EPLIN expression in the pancreatic cancer TCGA dataset. Normal samples: *n* = 4, median = 52.34, q1 = 42.79, q3 = 57.17; Tumour samples: *n* = 178, median = 73.46, q1 = 54.1, q3 = 102.59; *p* > 0.05. (**C**) EPLIN expression in comparison to differentiation in the TCGA pancreatic cancer dataset. Normal samples: *n* = 4, median = 52.34, q1 = 42.79, q3 = 57.17; Well differentiated samples: *n* = 31, median = 54.94, q1 = 35.1, q3 = 75.15; Moderate differentiated samples: *n* = 95, median = 73.138, q1 = 55.5, q3 = 102.44; Poor differentiated samples: *n* = 48, median = 86.13, q1 = 57.1, q3 = 112.1; Moderate differentiated samples vs. well differentiated samples, *p* = 0.02. Poor differentiated samples vs. well differentiated samples, *p* = 0.03. Box plot data shown is median expression, q1 and q3 values from each dataset, whiskers represent 5th and 95th percentiles with outliers shown. * represents *p* < 0.05, *** represents *p* < 0.001. Data from Fig. 1B&C was obtained from the UALCAN platform^30^ (https://ualcan.path.uab.edu/).

Public databases were also investigated to obtain the transcript expression profile of EPLIN in pancreatic cancer (Fig. 1). As Fig. 1A indicates, by exploring a pancreatic cancer GEO dataset (DGS4102), EPLIN transcript expression was significantly higher in tumour samples compared to normal samples (*p* < 0.001). Investigation of the TCGA dataset returned a similar result, however it did not reach statistical significance (Fig. 1B). Moreover, as Fig. 1C demonstrates, in the TCGA dataset, tumour samples that are classified as moderately differentiated (*p* = 0.02) and poorly differentiated (*p* = 0.03) had significantly higher EPLIN expression than well differentiated ones.

**Fig. 2:**
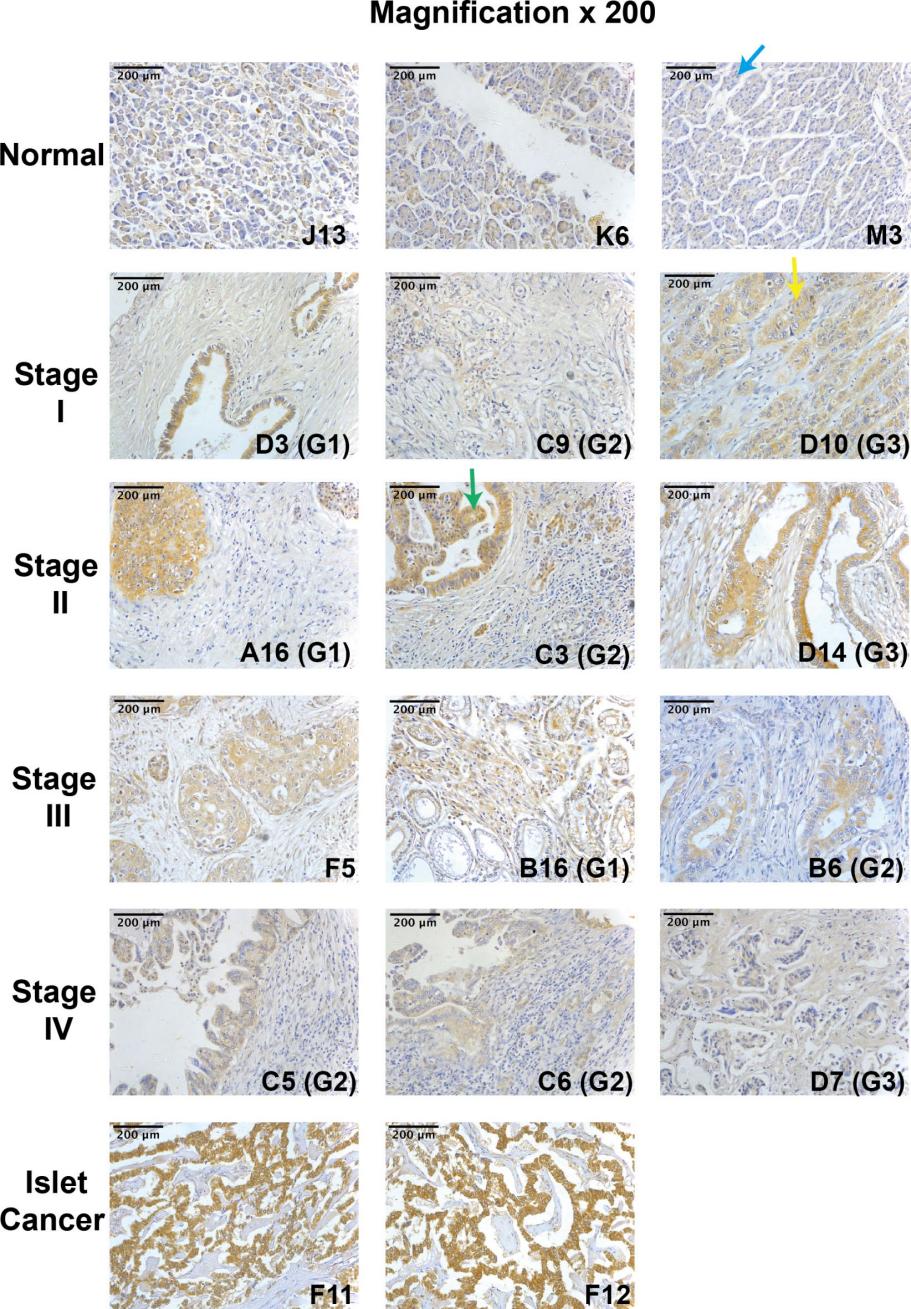
Representative photos of the TMA (PA2081a) stained with EPLIN. IHC was used for processing the TMA slide and probing EPLIN (sc-136399). EPLIN mainly distributes in cytoplasm (green arrow) The intensity of EPLIN’s protein expression was shown in brown colour (yellow arrow), while blue indicates negative expression of EPLIN (blue arrow). G1, G2 and G3 represent the grade levels. Photographs were taken under a Lecia DM IRB Microscope (Leica GmbH, Bristol, UK) at X200 objective magnification.

A TMA slide (PA2081a) was utilised to assess EPLIN expression at protein level using IHC analysis (Table 2; Fig. 2). As Fig. 2 shows, EPLIN was mainly stained in the cytoplasm in normal and pancreatic cancer tissues. As Table 2 shows, staining of EPLIN in tumour tissues was stronger than in normal tissue. Chi-square analysis among all the tissue types returned a significant difference (*p* < 0.001). Additionally, as the representative pictures (Fig. 2) demonstrate, the staining of EPLIN was generally stronger in tumour samples when compared to normal samples. For instance, the staining of EPLIN in stage I adenocarcinoma tissue (D10) was stronger than in the normal tissue (J13). The staining of EPLIN was also scored, classified and analysed based on tumour stage and differentiation status. However, no statistical significance was noted. Hence, EPLIN expression in tumour tissues was higher than in normal tissues in transcript and protein level.

**Table 1:** Transcript expression profile of EPLIN in comparison to clinical pathological information in the pancreatic cancer clinical cohort. Data is shown in mean ± SD. Two tailed T test was utilised to examinate the statistical significance.

Characteristic	Sample number (*n*)	Relative transcript expression (mean ± SD)	*p* - value
Tissue type			
Tumour	199	3.633 ± 0.898	
Normal	146	53,960,404 ± 49,210,036	0.27
Gender			
Male	120	3.59 ± 1.24	
Female	79	3.70 ± 1.27	0.95
Differentiation			
High	12	1.79 ± 1.77	
Moderate	68	1.99 ± 1.06	0.93
Low	12	1.88 ± 1.47	0.97
High and moderate	16	1.42 ± 0.79	
Moderate and low	68	6.5 ± 2.1	0.025
TNM stage			
TNM1	20	0.93 ± 0.59	
TNM2	126	3.7 ± 1.1	0.031
TNM3	18	5.3 ± 4.7	0.37
TNM4	11	3.4 ± 3.4	0.49
TNM1&2	32	3.3 ± 0.96	
TNM3&4	133	4.6 ± 3.1	0.70
T stage			
T1	5	5.6 ± 4.8	
T2	27	0.77 ± 0.45	0.37
T3	111	4.3 ± 1.3	0.80
T4	22	4.3 ± 3.8	0.84
T1&2	32	1.53 ± 0.84	
T3&4	133	4.3 ± 1.2	0.064
Nodal involvement			
Negative	80	3.4 ± 1.3	
Positive	99	3.9 ± 1.4	0.79
Presence of metastases			
No metastasis	184	3.6 ± 0.94	
Distant metastasis	15	4.2 ± 2.9	0.84
Vascular embolism			
Negative	114	3.3 ± 1.1	
Positive	55	3.4 ± 1.7	0.94
Survival			
Alive	44	4.1 ± 2.3	
Died	139	3.8 ± 1.1	0.91

**Table 2:** Scoring analysis of the pancreatic cancer TMA (PA2081a).

	Total Number	Intensity		Statistical significance	
		Negative to weak (0–1)	Moderate to strong (2–3)	Chi value	*p*
Pathology				24.38	< 0.001^a^
Normal tissue	20	10	10		
Cancer adjacent normal tissue	42	26	16		
Adenosquamous carcinoma	6	1	5		
Ductal adenocarcinoma	84	27	57		
Islet cell carcinoma	22	1	21		
Stage				2.034	0.5654^b^
I	44	15	29		
II	30	9	21		
III	12	2	10		
IV	4	2	2		
Differentiation code				4.419	0.1097^c^
Grade1	14	2	12		
Grade2	25	12	13		
Grade3	34	13	21		

Note: ^a^Overall chi-square test among pathology groups; ^b^Overall chi-square test among stage groups; ^c^Overall chi-square test among differentiation groups. Well differentiation (G1), moderate differentiation (G2) and poor differentiation (G3).

### High expression of EPLIN leads to poor clinical outcomes in pancreatic cancer

**Fig. 3:**
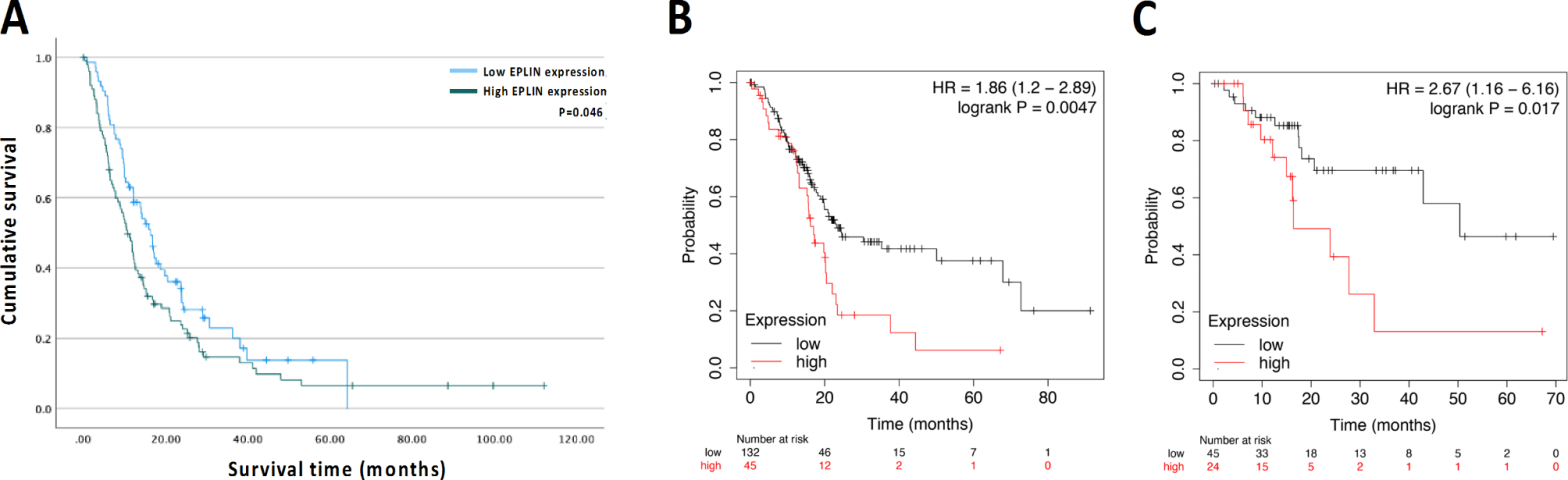
Kaplan-Meier survival analysis of EPLIN in pancreatic cancer. (**A**) EPLIN transcript expression on patient’s overall survival in the pancreatic cancer clinical cohort. High EPLIN expression: *n* = 101, mean survival = 20.02 months, 95%CI: 14.14–25.89; Low EPLIN expression: *n* = 73, mean survival = 22.63 months, 95%CI: 17.56–27.70; *p* = 0.046. (**B**) Implication of EPLIN transcript expression on patient’s overall survival in the TCGA pancreatic cancer dataset. High EPLIN expression: *n* = 45, median survival = 16.17 months; Low EPLIN expression: *n* = 132, median survival = 23.17; *p* = 0.0047. (**C**) Implication of EPLIN transcript expression on patient’s relapse-free survival (RFS) in the TCGA pancreatic cancer dataset. High EPLIN expression: *n* = 24, median survival = 16.4 months; Low EPLIN expression: *n* = 45, median survival = 50.37 months; *p* = 0.017. Survival data of the pancreatic cancer TCGA dataset was analysed and obtained from the Kaplan-Meier Plotter (https://kmplot.com/).

The pancreatic cancer clinical cohort was analysed to conduct Kaplan-Meier survival analysis. As Fig. 3A shows, patients with high transcript expression of EPLIN had a significantly worse overall survival (OS) than patients with low transcript expression of EPLIN (*p* = 0.046). Similarly, investigating the TCGA dataset on Kaplan-Meier Plotter^31^ returned a consistent result. As Fig. 3B demonstrates, patients with high EPLIN expression had a worse OS than those with low EPLIN expression (*p* = 0.0047). While high EPLIN expression also shortened patient’s relapse-free survival (RFS) in the TCGA dataset (*p* = 0.0017) (Fig. 3C). Therefore, high EPLIN expression is related to poor OS and RFS in pancreatic cancer patients.

### EPLIN positively impacts on cellular growth and migration in pancreatic cancer

**Fig. 4:**
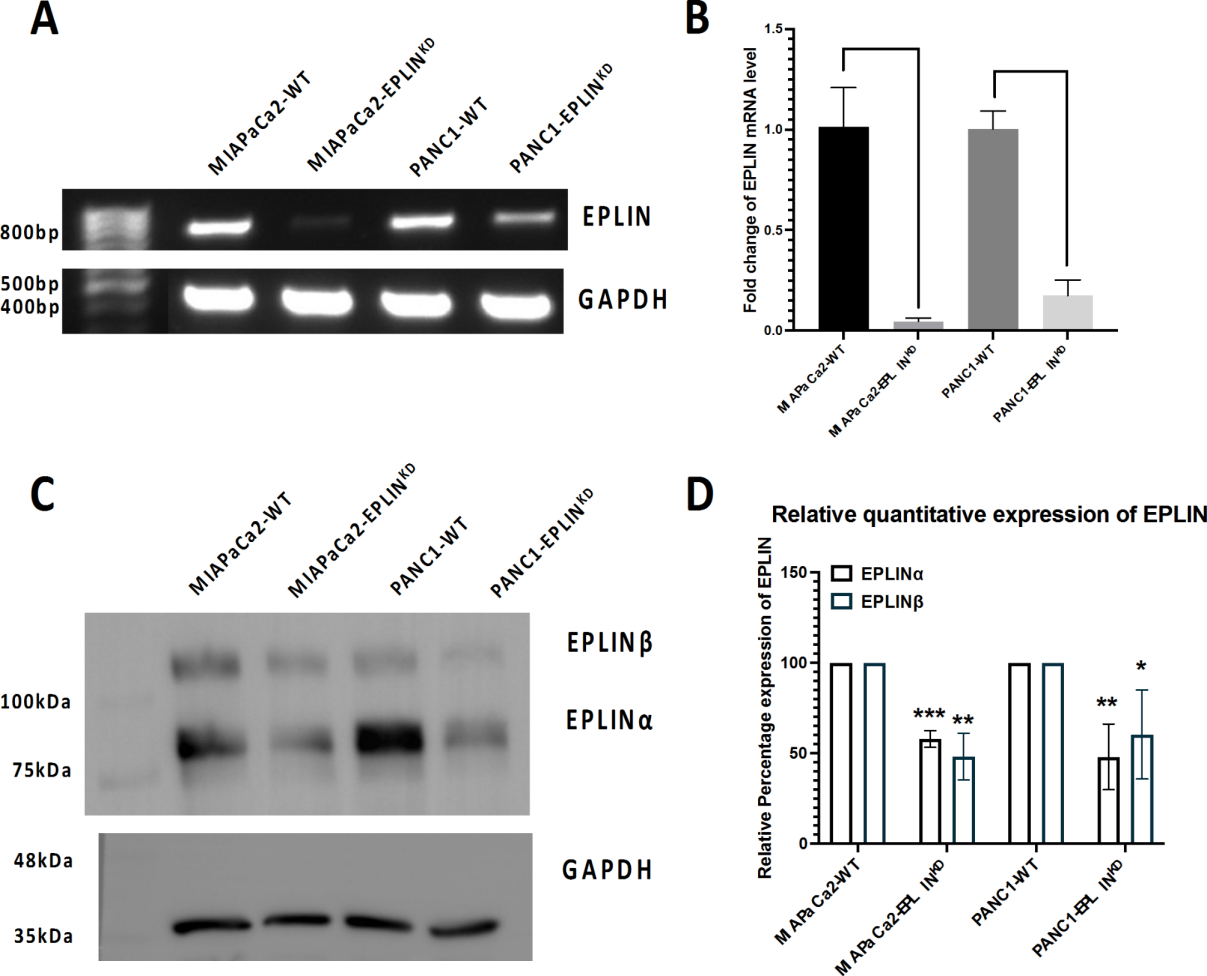
Generation of EPLIN knockdown models in pancreatic cancer cell lines. (**A**) Conventional PCR shows the mRNA level of EPLIN was downregulated in knockdown models compared to their control wild type (WT) cell line respectively. (**B**) qPCR indicates EPLIN was knocked down significantly in MIAPaCa2 cell line (*n* = 3, *p* = 0.001) and PANC1 cell line (*n* = 3, *p* < 0.001). Data was shown at mean ± SD. (**C**) Representative figures of Western Blotting (WB) showing EPLIN was knocked down in both pancreatic cancer cell lines at protein level. (**D**) Semi-quantification of the WB screening probing EPLIN (*n* = 3). Normalised expression of EPLIN in WT groups were used as reference to calculate relative expression in KD group. Percentage change: EPLINβ: MIAPaCa2-WT = 100; MIAPaCa2-EPLIN^KD^=47.9 ± 18.2, *p* = 0.002; PANC1-WT = 100; PANC1-EPLIN^KD^= 67.9 ± 29.4, *p* = 0.049. EPLINα: MIAPaCa2-WT = 100; MIAPaCa2-EPLIN^KD^ =57.3 ± 6.4, *p* < 0.001; PANC1-WT = 100; PANC1-EPLIN^KD^ = 52.7 ± 22.9, *p* = 0.0077. Intensity ratio was normalised by GAPDH. Data was shown at mean. * represents *p* < 0.05, ** represents *p* < 0.01, *** represents *p* < 0.001. Files of the unprocessed PCR gel electrophoresis result was shown in S1. Replicated original WB screening were demonstrated in S2 and S10.

Two pancreatic cancer cell lines, MIAPaCa2 and PANC1, were used to establish cell models, by manipulating EPLIN expression. As Fig. 4 indicates, EPLIN was successfully knocked down in both cell lines at RNA level, as assessed by conducting conventional PCR (Fig. 4A) and qPCR (Fig. 4B). Western blotting was also performed to confirm EPLIN was knocked down in both cell lines at the protein level (Fig. 4C&D).

The two established pancreatic cancer cell lines models were used to carry out cellular growth assay. After three days, as Fig. 5A demonstrates, knocking down EPLIN resulted in reduced cellular growth rates in both models. Compared to the control MIAPaCa2 model, MIAPaCa2-EPLIN^KD^ cells showed a significantly decreased growth rate by 42.2% (*p* < 0.001). Similarly, in the PANC1 models, knocking down EPLIN led to a significant 20% lower growth rate against the control group (*p* = 0.0047).

EPLIN has been identified as a profound negative regulator of cellular migration in cancer cells in the past decades^16,20,23,24^. Therefore, we investigated EPLIN’s impact on migration by applying the cell models to ECIS and wound scratching assays. As Fig. 5B shows, by conducting ECIS based migration assays, the knockdown model resulted in reduced cellular migration compared to the control group, in MIAPaCa2 models. The 3D model of both MIAPaCa2-WT (Fig. 5B middle panel) and MIAPaCa2-EPLIN^KD^ (Fig. 5B right panel) indicated more dynamic changes between the two models across different frequencies and time points. A similar effect was also observed within the PANC1 models (Fig. 5C). The changes of impedance could also be observed across different frequencies and time points, in the 3D models of PANC1-WT (Fig. 5C middle panel) and PANC1-EPLIN^KD^ (Fig. 5C right panel). Additionally, wound scratching assays on the in vitro models demonstrated a similar result (Fig. 5D&E). In MIAPaCa2 models, EPLIN knockdown resulted in significant decrease of migration at all 4 recorded time points (all *p* < 0.05) (Fig. 5D). Similarly, inhibition of EPLIN also led to slower migration in PANC1 models (all *p* < 0.05) (Fig. 5E). Hence, EPLIN regulates cellular growth and migration positively in pancreatic cancer cells.

**Fig. 5:**
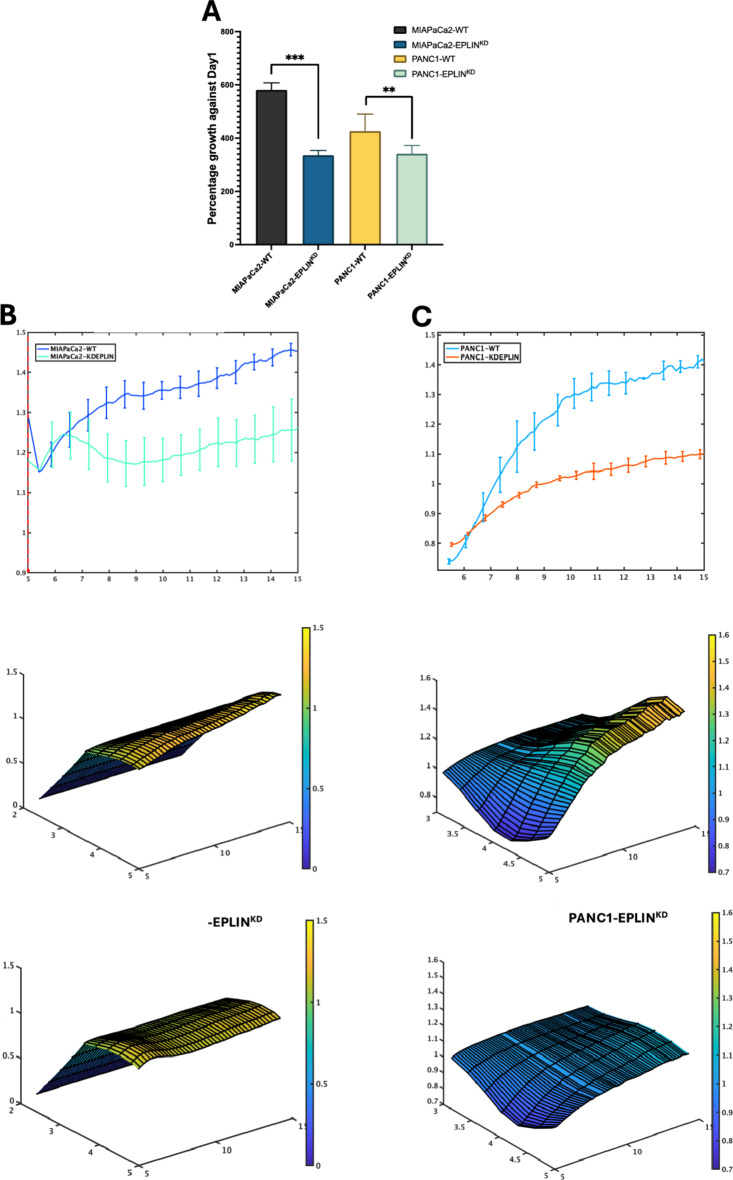
EPLIN acts as a positive regulator of cellular growth and migration in pancreatic cancer. (**A**) MTT based cellular growth assay showing downregulation of EPLIN led to reduced growth rates against Day1. MIAPaCa2-WT: 580.7 ± 27.1, *n* = 8; MIAPaCa2-EPLIN^KD^: 335.8 ± 18.0, *n* = 8, *p* < 0.001. PANC1-WT: 426.2 ± 64.5, *n* = 8; PANC1-EPLIN^KD^: 341.0 ± 31.4, *n* = 8, *p* = 0.0047. Data was shown at mean ± SD. (**B**) ECIS assays on MIAPaCa2 models. The left panel: recorded normalised impendence in MIAPaCa2 models at 8000 Hz for 10 h. The middle panel: 3D model of the MIAPaCa2-WT group. The right panel: 3D model of the MIAPaCa2-EPLIN^KD^ group. (**C**) ECIS assays on PANC1 models. The left panel: recorded normalised impendence in PANC1 models at 8000 Hz for 10 h. The middle panel: 3D model of the PANC1-WT group. The right panel: 3D model of the PANC1-EPLIN^KD^ group. Impendence data was normalised based on the raw data at the electrical wounding point (mean ± SD). (**D**) Wound scratching assay for MIAPaCa2 models. Mean closed area (pixel): Hour-1: MIAPaCa2-WT: 14253.3 ± 3227.9, MIAPaCa2-EPLIN^KD^: 6403 ± 2053.3, *p* = 0.024. Hour-2: MIAPaCa2-WT: 28,013 ± 7451.5, MIAPaCa2-EPLIN^KD^: 11763.3 ± 6371.7, *p* = 0.045. Hour-3: MIAPaCa2-WT: 44,182 ± 7251.8, MIAPaCa2-EPLIN^KD^: 17119.7 ± 6068, *p* = 0.008. Hour-4: MIAPaCa2-WT: 56142.7 ± 6329.4, MIAPaCa2-EPLIN^KD^: 26978.7 ± 12071.3, *p* = 0.021. *n* = 3. Representative pictures of each model at both hour-0 and hour-4 are shown on the right. (**E**) Wound scratching assay for PANC1 models. Mean closed area (pixel): Hour1: PANC1-WT: 22861.3 ± 6427, PANC1-EPLIN^KD^: 10772.7 ± 2379.8, *p* = 0.038. Hour-2: PANC1-WT: 51143.3 ± 8346.3, PANC1-EPLIN^KD^: 31902.7 ± 5910.1, *p* = 0.031. Hour-3: PANC1-WT: 69118.3 ± 9894.8, PANC1-EPLIN^KD^: 43628.7 ± 8200, *p* = 0.026. *n* = 3. Hour-4: PANC1-WT: 93,495 ± 11797.7, PANC1-EPLIN^KD^: 56,620 ± 12078.1, *p* = 0.019. Representative pictures of each model at both hour-0 and hour-4 are shown on the right. Data was shown at mean ± SD. * represents *p* < 0.05, ** represents *p* < 0.01, *** represents *p* < 0.001.

**Fig. 5:**
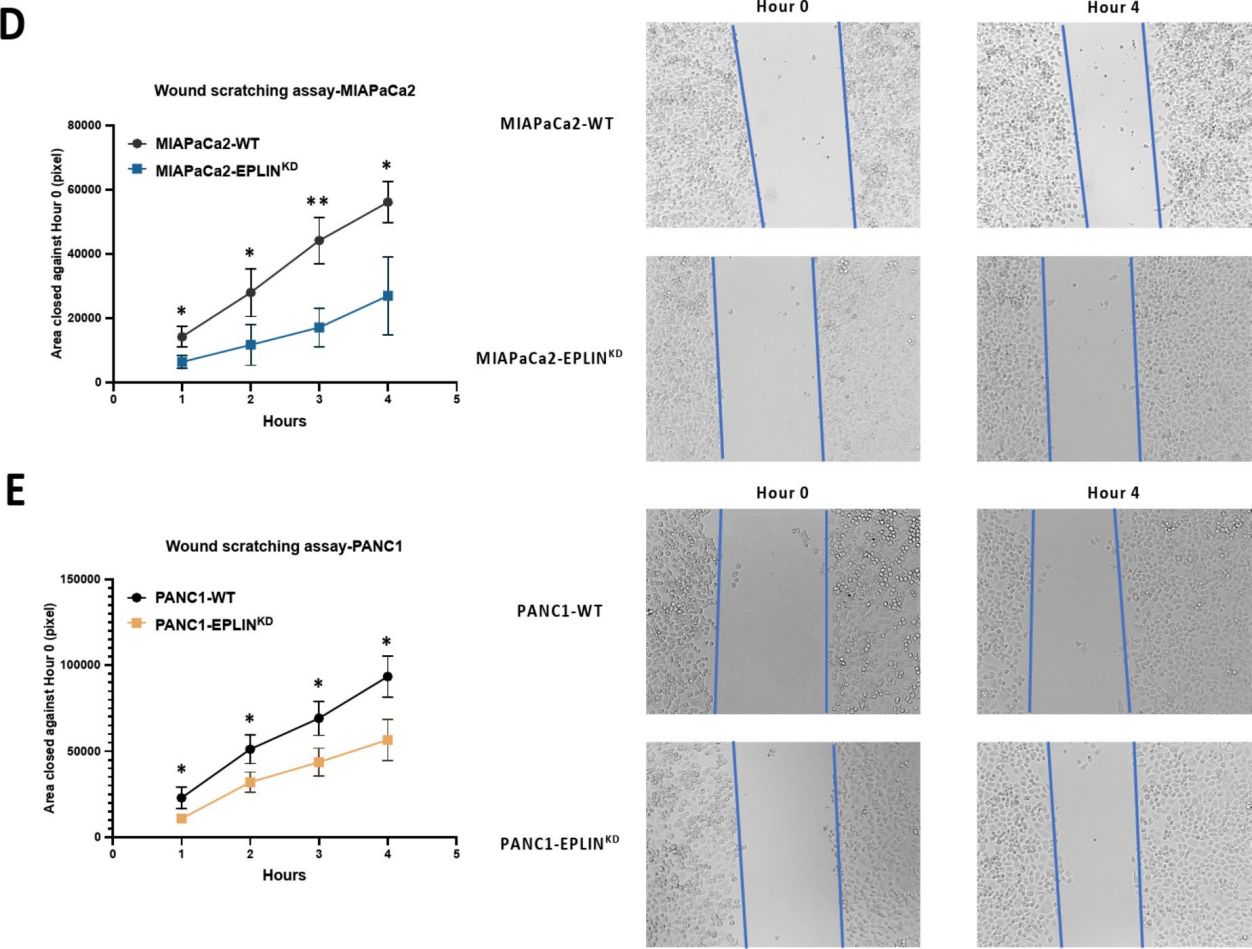
(continued)

### EPLIN promotes cell response to chemotherapeutic and EGFR/HER2 targeted therapeutic agents in pancreatic cancer

**Fig. 6:**
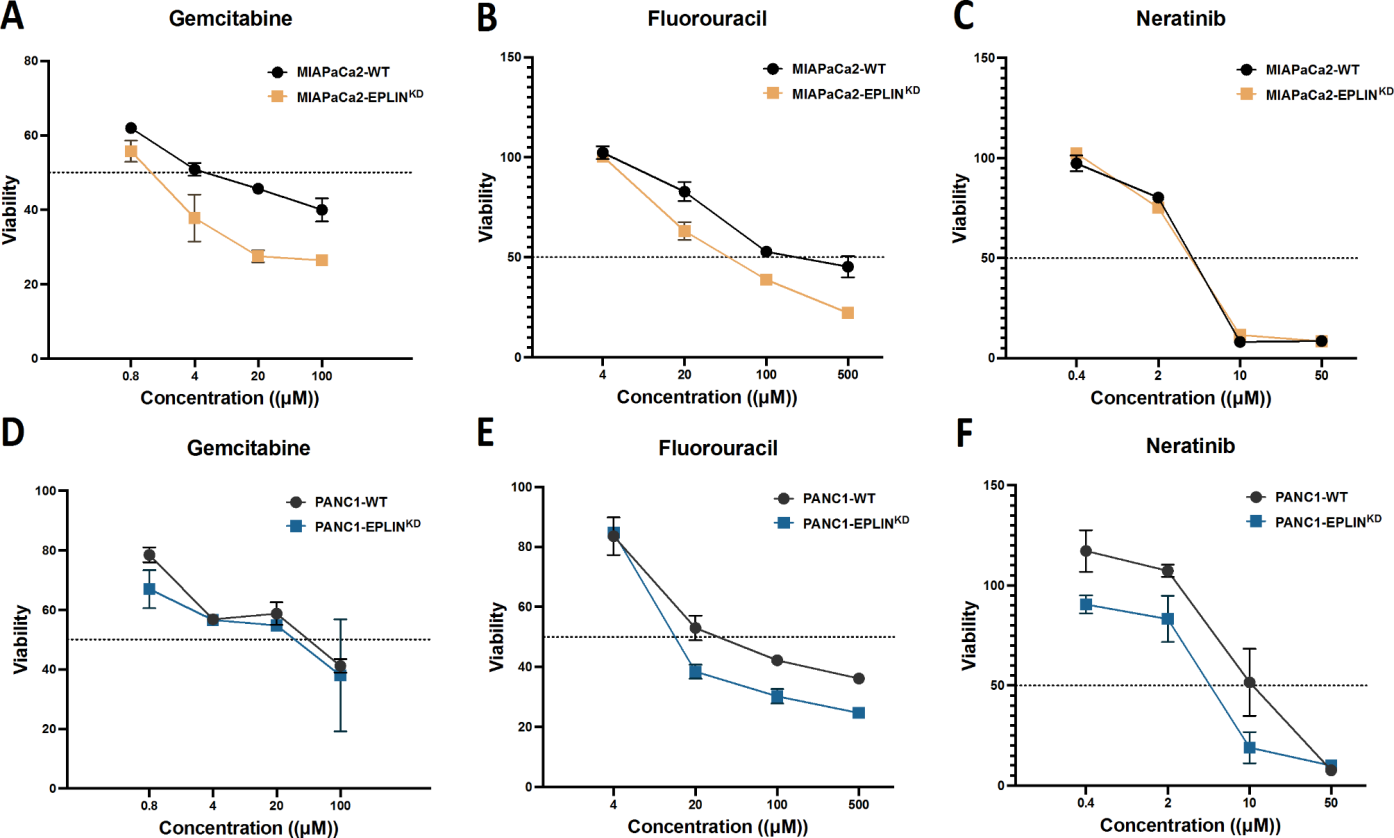
EPLIN has an impact on pancreatic cancer cell response to chemotherapeutic and EGFR/HER2 targeted therapeutic agents. (**A**) Gemcitabine cytotoxicity assays on MIAPaCa2 models. (**B**) Fluorouracil (5FU) cytotoxicity assays on MIAPaCa2 models. (**C**) Neratinib cytotoxicity assays on MIAPaCa2 models. (**D**) Gemcitabine cytotoxicity assays on PANC1 models. (**E**) Fluorouracil (5FU) cytotoxicity assays on PANC1 models. (**F**) Neratinib cytotoxicity assays on PANC1 models. Therapeutic agents were serial diluted (gemcitabine: 0.8–100µM, 5FU: 4-500µM, neratinib: 0.4–50µM) to apply on the assays. *n* = 3, data was shown at mean ± SD.

The established pancreatic EPLIN manipulated models were also used to perform cytotoxicity assays, with serial diluted therapeutic agents, to explore its influence on drug resistance (Fig. 6; Table 3). Firstly, EPLIN knockdown led to more sensitive response to gemcitabine and fluorouracil (5FU) in MIAPaCa2 models, as Fig. 6A&B and Table 3 indicated. A similar trend was also observed in PANC1 models, where the EPLIN knockdown group resulted in lower IC50s to these two key therapeutic agents for pancreatic cancer compared to the control group (Fig. 6D&E and Table 3). In the case of neratinib, an inhibitor for EGFR/HER2, EPLIN knockdown led to more sensitive response in the PANC1 models, whereas such impact was not observed in the MIAPaCa2 models. Hence EPLIN was demonstrated to have a potential to enhance pancreatic cancer cells’ resistances to two chemotherapeutic agents, gemcitabine and 5FU. It also has a similar impact on neratinib in PANC1 cells.

**Table 3:** Predicted IC50 values of cytotoxicity assays. IC50s was calculated based on the logarithmic trend line.

	MIAPaCa2-WT	MIAPaCa2-EPLIN^KD^	PANC1-WT	PANC1-EPLIN^KD^
Gemcitabine	7.0 µM	1.3 µM	31.0 µM	17.0 µM
5FU	237.0 µM	65.5 µM	42.3 µM	21.4 µM
Neratinib	4.2 µM	4.3 µM	5.5 µM	2.2 µM

### EPLIN regulates expression levels of key components in MAPK and PIK3CA-AKT signalling events and EMT

Given that the pancreatic cancer cell lines applied in the current study both harboured *KRAS* mutation, the most frequent genetic mutation observed in pancreatic cancer, we were interested in investigating if EPLIN is involved in the KRAS networks. Pearson correlation was conducted between EPLIN and some key players in KRAS-activated signalling events in the pancreatic cancer TCGA database (Table 4). mRNA level of EPLIN was found to be positively correlated with 3 out of 4 members of the EGFR family significantly, namely EGFR *(p* < 0.0001*)*, ERBB2 (HER2) (*p* < 0.0001) and ERBB3 (HER3) (*p* < 0.0001). As the table shows, its mRNA level also positively correlates with PIK3CA significantly (*p* = 0.0002), a crucial downstream effactor of KRAS in pancreatic cancer. However, no significant correlation was observed among the key genes in PIK3CA-AKT signalling, apart from PRKCA *(p* < 0.0001*)*, a downstream effector that was previously reported to be overexpressed in pancreatic cancer^32^. Furthermore, another essential protein with parallel signalling after activation of KRAS, MAPK, was also tested. The mRNA level of all key components was returned a significant correlation with EPLIN, such as KRAS (*p* < 0.0001), BRAF (*p* = 0.0002), RAF1 (*p* < 0.0001), MAP2K1 (MEK1) (*p* = 0.0158), MAPK1 (ERK1) (*p* = 0.0059) and MAPK3 (ERK2) (*p* < 0.0001). However, rather than positive correlation, some genes have significant negative correlation with EPLIN in this pathway.

**Fig. 7:**
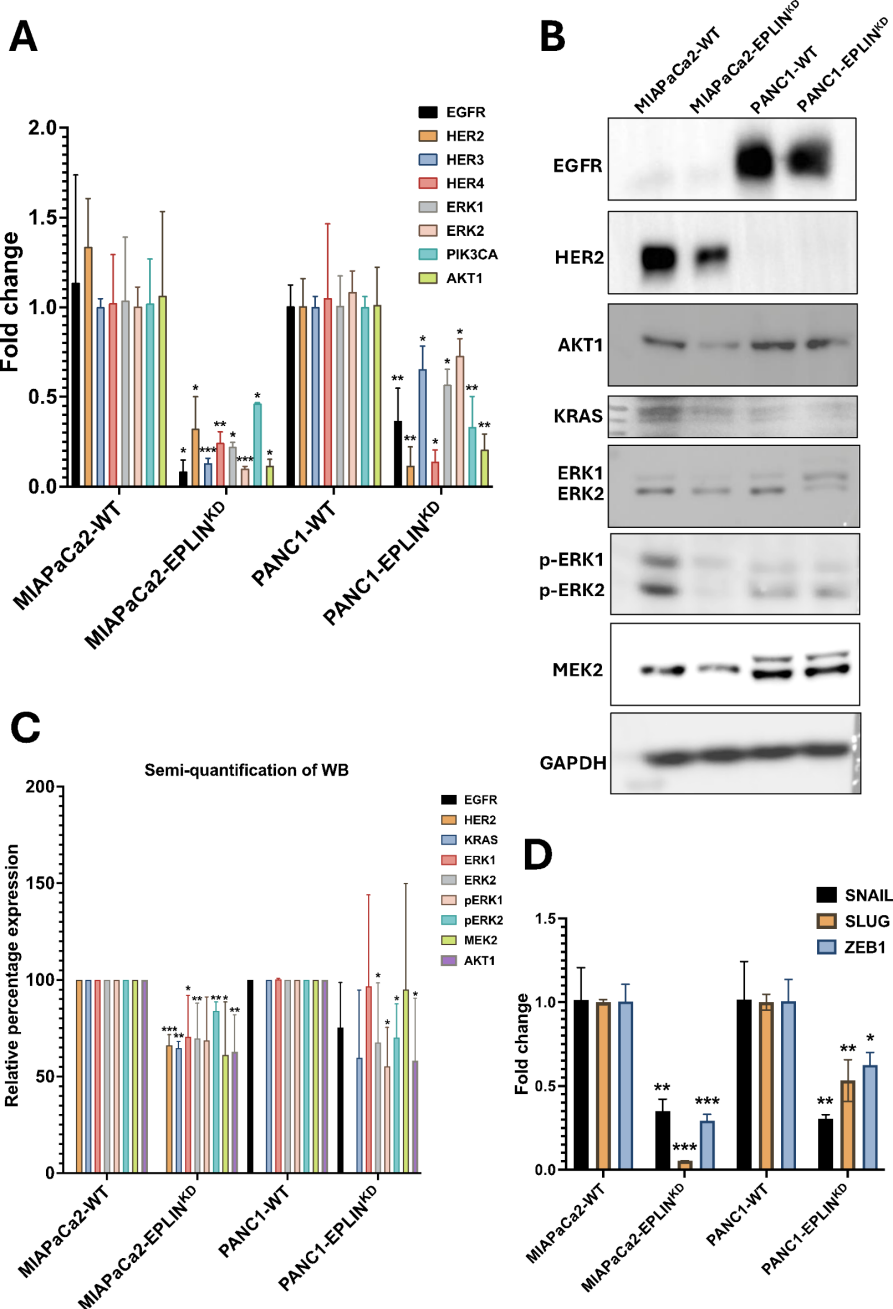
EPLIN regulates expressions of several key players in MAPK and PIK3CA-AKT signalling pathways, as well as in EMT. (**A**) qPCR results of probing RTK family and several key components in MAPK&PIK3CA-AKT signalling events in the pancreatic cancer cell models. (**B**) Representative western blotting screening showing protein expression levels of several key components in the signalling pathways in both MIAPaCa2 and PANC1 cell models. (**C**) Semi-quantification of the western blotting results (*n* ≥ 3). relative percentage change: EGFR: PANC1-EPLIN^KD^vs. PANC1-WT = 75.5 ± 23.2, *p* = 0.14; HER2: MIAPaCA2-EPLIN^KD^vs. MIAPaCa2-WT = 66.2 ± 5.5, *p* < 0.001; AKT1: MIAPaCA2-EPLIN^KD^vs. MIAPaCa2-WT = 62.9 ± 19.2, *p* = 0.0082, PANC1-EPLIN^KD^vs. PANC1-WT = 58.2 ± 32.2, *p* = 0.041; KRAS: MIAPaCA2-EPLIN^KD^vs. MIAPaCa2-WT = 64.85 ± 3.3, *p* < 0.001, PANC1-EPLIN^KD^vs. PANC1-WT = 45.5 ± 34.8, *p* = 0.16; ERK1: MIAPaCA2-EPLIN^KD^vs. MIAPaCa2-WT = 56.3 ± 11.8, *p* = 0.015, PANC1-EPLIN^KD^vs. PANC1-WT = 108.68 ± 45.1, *p* = 0.87; ERK2: MIAPaCA2-EPLIN^KD^vs. MIAPaCa2-WT = 70.0 ± 18.3, *p* = 0.0062, PANC1-EPLIN^KD^vs. PANC1-WT = 67.7 ± 30.8, *p* = 0.047; p-ERK1: MIAPaCA2-EPLIN^KD^vs. MIAPaCa2-WT = 68.7 ± 22.5, *p* = 0.074, PANC1-EPLIN^KD^vs. PANC1-WT = 55.4 ± 20.0, *p* = 0.018; p-ERK2: MIAPaCA2-EPLIN^KD^vs. MIAPaCa2-WT = 84.0 ± 4.6, *p* = 0.0039, PANC1-EPLIN^KD^vs. PANC1-WT = 70.1 ± 17.5, *p* = 0.042; MEK2: MIAPaCA2-EPLIN^KD^vs. MIAPaCa2-WT = 61.2 ± 27.4, *p* = 0.013, PANC1-EPLIN^KD^vs. PANC1-WT = 60.2 ± 38.1, *p* = 0.048. Expression levels of proteins were normalised based on GAPDH. ImageJ and GraphPad were used for the semi-quantification. (**D**) qPCR results of probing RTK family and several key contributors to EMT. Data was shown at mean ± SD, * represents *p* < 0.05, ** represents *p* < 0.01, *** represents *p* < 0.001. Files of the replicated original WB screening were demonstrated in S3 to S10.

Apart from exploring the TCGA public database, qPCR was carried out in the cell models to assess the mRNA levels of some of the key contributors mentioned above (Fig. 7A). As the figure demonstrated, in the MIAPaCa2 cell models, inhibition of EPLIN led to significant decrease of all four members of RTK family (EGFR: *p* = 0.039, HER2: *p* = 0.014, HER3: *p* < 0.001, HER4: *p* = 0.008). ERK1 (*p* = 0.016) and ERK2 (*p* = 0.0001) were also downregulated in the knockdown model compared to the WT group. When it comes to the components in PI3KCA-AKT1 signalling event, two key players, knocking down EPLIN resulted in significant downregulation of PIK3CA (*p* = 0.018) and AKT1 (*p* = 0.025). Similar effects were observed in the PANC1 models, followed by the inhibition of EPLIN, downregulation of RNA levels of EGFR (*p* = 0.0069), HER2 (*p* = 0.0059), HER3 (*p* = 0.013), HER4 (*p* = 0.019), ERK1 (*p* = 0.015), ERK2 (*p* = 0.032), PIK3CA (*p* = 0.0029) and AKT1 (*p* = 0.0034) were observed.

Moreover, the protein levels of some key players in such signalling pathways were also accessed by carrying out western blotting in the established cell models (Fig. 7B&C). To begin with, no statistical significance was noted in regards to EGFR after inhibiting EPLIN in the cell models (*p* > 0.05). Another RTK family member, HER2 was also probed. Although its protein level was too low to be observed in the PANC1 models. A significant downregulated level of HER2 was noted after knocking down in the MIAPaCa2 cells (*p* = 0.00045). Secondly, inhibition of EPLIN resulted in downregulation of KRAS significantly in MIAPaCa cell lines (*p* < 0.001), similar trend was observed in the PANC1 models, however, non-significance was noted. Thirdly, the expression level of AKT1, an essential downstream effector of PIK3CA-AKT signalling pathway, was downregulated following by knocking down EPLIN in both MIAPaCa2 (*p* = 0.0082) and PANC1 cells (*p* = 0.041). Moreover, key proteins of the parallel signalling event activated by KRAS, MAPK, were tested. Comparing to the WT group, expressions of ERK1 (*p* = 0.017) and ERK2 (*p* = 0.0062) were downregulated when EPLIN was inhibited in MIAPaCa2 cells. No change of ERK1, but ERK2 (*p* = 0.047) was observed in the PANC1 models. Interestingly, following the inhibition of EPLIN, the phosphorylation level of ERK2 was downregulated significantly in MIAPaCa2 cells (*p* = 0.0039), a near significant decrease of pERK1 was also noted (*p* = 0.074).The phosphorylation levels of both ERK1 (*p* = 0.018) and ERK2 (*p* = 0.042) were noted to decrease after inhibiting EPLIN in PANC1 cell lines. Besides, the upstream of ERK1/2, MEK2 was also observed to decrease significantly in the EPLIN knocked down MIAPaCa2 cells compared to the WT group (*p* = 0.013). Collectively, we observed EPLIN positively regulate protein expression several key proteins in MAPK and PIK3CA-AKT signalling in our pancreatic cancer cell modes. Such results were largely in line with the change of mRNA level in the TCGA dataset. EPLIN also related to the phosphorylation of ERK1/2. Hence, EPLIN might act as an important upstream regulator of MAPK and PIK3CA-AKT signalling events in pancreatic cancer.

EPLIN has been reported as a negative regulator of EMT in several cancer types by regulating several contributors, such as SNAIL, SLUG and ZEB1/2^16,18^. Here we revealed the regulatory relationship between EPLIN and the these EMT contributors in pancreatic cancer cells. By performing qPCR analysis on our EPLIN inhibited models, we accessed the RNA expression levels of SNAIL, SLUG and ZEB1/2 (Fig. 7D). As the figure indicated, inhibition of EPLIN led to a significant downregulation of SNAIL in both cell models (MIAPaCa2: *p* = 0.0052, PANC1:*p* = 0.0057). EPLIN also regulated SLUG positively (MIAPaCa2: *p* < 0.001, PANC1:*p* = 0.0037). Similarly, ZEB1 was downregulated significantly followed by the diminish of EPLIN (MIAPaCa2: *p* < 0.001, PANC1:*p* = 0.012).

**Table 4:** Correlation between EPLIN and components in key KRAS activated signalling in the TCGA pancreatic cancer cohort (*n* = 183).

	Pearson *R*	95% confidence interval	*P* value
PIK3CA signalling			
PIK3CA	0.2709	0.1310 to 0.4003	0.0002
MTOR	0.007765	-0.1374 to 0.1526	0.9169
AKT1	0.0395	-0.1062 to 0.1835	0.5955
MYC	0.117	-0.02857 to 0.2577	0.1148
PRKCA	0.328	0.1921 to 0.4516	< 0.0001
PRKCB	0.03425	-0.1114 to 0.1784	0.6453
MAPK signalling			
KRAS	0.5504	0.4405 to 0.6440	< 0.0001
BRAF	0.2686	0.1285 to 0.3981	0.0002
RAF1	0.2988	0.1607 to 0.4254	< 0.0001
MAP2K1	0.1781	0.03396 to 0.3151	0.0158
MAP2K2	-0.3779	-0.4958 to -0.2463	< 0.0001
MAP2K3	0.214	0.07116 to 0.3483	0.0036
MAP2K4	-0.1625	-0.3004 to -0.01782	0.028
MAP2K5	-0.2718	-0.4011 to -0.1320	0.0002
MAP2K6	-0.163	-0.3010 to -0.01840	0.0275
MAP2K7	-0.3177	-0.4423 to -0.1809	< 0.0001
MAPK1	0.2027	0.05940 to 0.3378	0.0059
MAPK3	0.4334	0.3076 to 0.5442	< 0.0001
Receptor tyrosine kinase (RTK) family			
EGFR	0.3336	0.1982 to 0.4566	< 0.0001
ERBB2	0.3713	0.2391 to 0.4900	< 0.0001
ERBB3	0.4458	0.3215 to 0.5549	< 0.0001
ERBB4	-0.0806	-0.2242 to 0.06645	0.2821

### PIK3s, ERK and MEK’s inhibition requires the presence of EPLIN in cell migration

**Fig. 8:**
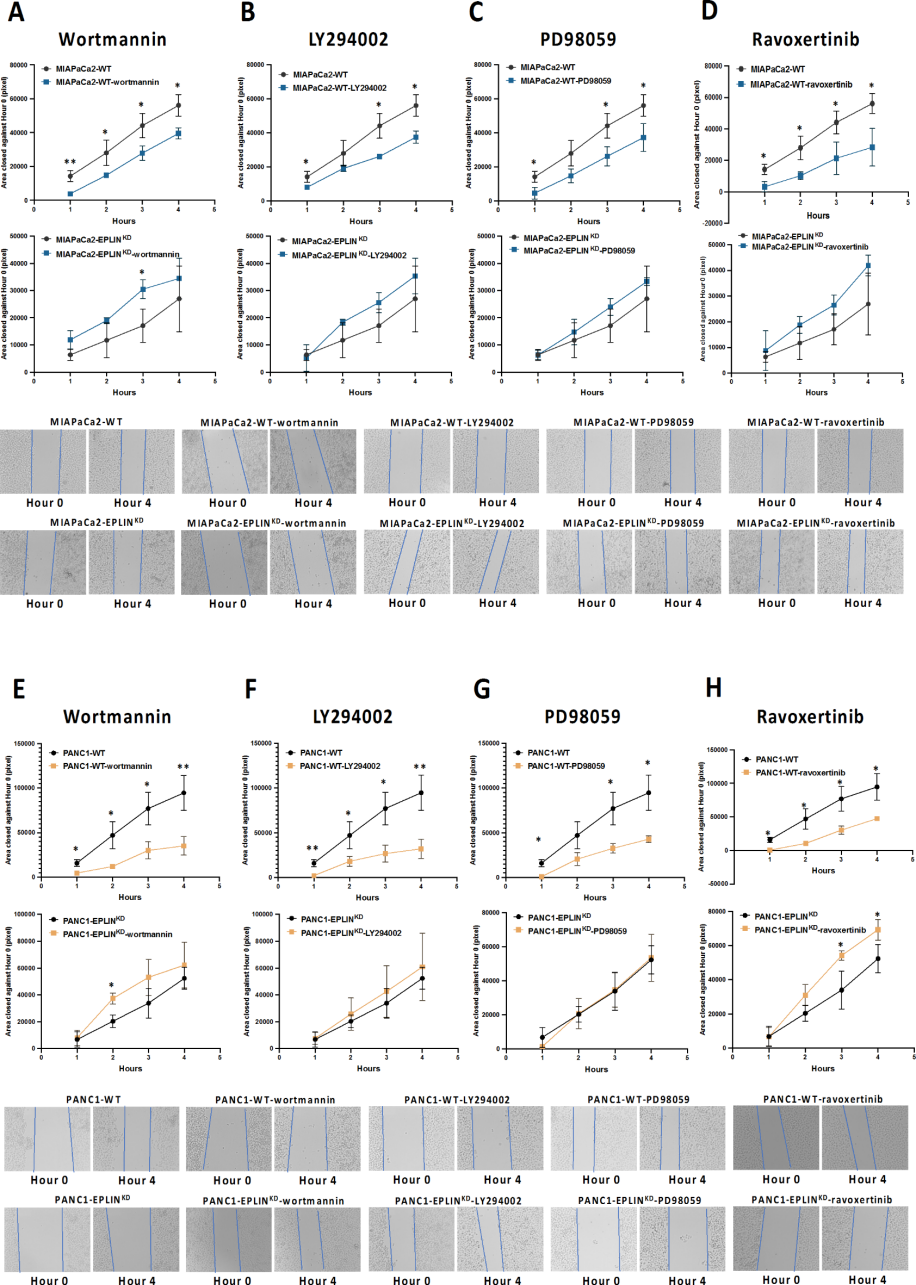
EPLIN mediates inhibitors of PIK3 kinases, ERK and MEK’s effects on pancreatic cancer cellular migration. (**A**) Wound scratching based migration assays on MIAPaCa2 models along with wortmannin. Mean closed area (pixel): Hour 1: MIAPaCa2-WT: 14253.3 ± 3227.9, MIAPaCa2-WT-wortmannin: 3873 ± 860.2, *p =* 0.006; MIAPaCa2-EPLIN^KD^: 6403 ± 2053.3, MIAPaCa2-EPLIN^KD^-wortmannin: 11914.7 ± 3336, *p* > 0.05; Hour 2: MIAPaCa2-WT: 28,013 ± 7451.5, MIAPaCa2-WT-wortmannin: 14,761 ± 658.5, *p =* 0.037; MIAPaCa2-EPLIN^KD^: 11763.3 ± 6371.6371.7, MIAPaCa2-EPLIN^KD^-wortmannin: 18,987 ± 1071.4, *p* > 0.05; Hour 3: MIAPaCa2-WT: 44,182 ± 7251.8, MIAPaCa2-WT-wortmannin: 27,850 ± 4242, *p* = 0.008; MIAPaCa2-EPLIN^KD^: 17,120 ± 6068, MIAPaCa2-EPLIN^KD^-wortmannin: 30511.7 ± 3434.4, *p* = 0.029; Hour 4: MIAPaCa2-WT: 56142.7 ± 6329.4, MIAPaCa2-WT-wortmannin: 39,564 ± 3262.2, *p* = 0.021; MIAPaCa2-EPLIN^KD^: 26978.7 ± 12071.3, MIAPaCa2-EPLIN^KD^-wortmannin: 34,446 ± 7458.8, *p* > 0.05. (**B**) Wound scratching based migration assays on MIAPaCa2 models along with LY294002. Mean closed area (pixel): Hour 1: MIAPaCa2-WT-LY294002: 8087 ± 663.1, *p* = 0.032; MIAPaCa2-EPLIN^KD^-LY294002: 5232.7 ± 4849, *p* > 0.05; Hour 2: MIAPaCa2-WT-LY294002: 19192.3 ± 1801.4, *p* > 0.05; MIAPaCa2-EPLIN^KD^-LY294002: 18,375 ± 1052.8, *p* > 0.05; Hour 3: MIAPaCa2-WT-LY294002: 26197.7 ± 789.4, *p* = 0.013; MIAPaCa2-EPLIN^KD^-LY294002: 25584.7 ± 3685.2, *p* > 0.05; Hour 4: MIAPaCa2-WT-LY294002: 37595.7 ± 3615.9, *p* = 0.012; MIAPaCa2-EPLIN^KD^-LY294002: 35352.3 ± 6529.5, *p* > 0.05. (**C**) Wound scratching based migration assays on MIAPaCa2 models along with PD98059. Mean closed area (pixel): Hour 1: MIAPaCa2-WT-PD98059: 4591 ± 3442.1, *p* = 0.024; MIAPaCa2-EPLIN^KD^-PD98059: 6383.7 ± 1755.3, *p* > 0.05; Hour 2: MIAPaCa2-WT-PD98059: 14,836 ± 4102.4, *p* > 0.05; MIAPaCa2-EPLIN^KD^-PD98059: 14,740 ± 4661.3, *p* > 0.05; Hour 3: MIAPaCa2-WT-PD98059: 26,292 ± 5550.7, *p* = 0.027; MIAPaCa2-EPLIN^KD^-PD98059: 23,976 ± 3060.4, *p* > 0.05; Hour 4: MIAPaCa2-WT-PD98059: 37,301 ± 8166, *p* = 0.034; MIAPaCa2-EPLIN^KD^-LY294002: 33,294 ± 1444.8, *p* > 0.05. (**D**) Wound scratching based migration assays on MIAPaCa2 models along with ravoxertinib. Mean closed area (pixel): Hour 1: MIAPaCa2-WT-ravoxertinib: 3254.3 ± 3328.7, *p* = 0.015; MIAPaCa2-EPLIN^KD^-ravoxertinib: 8807.7 ± 7743.2, *p* > 0.05; Hour 2: MIAPaCa2-WT-ravoxertinib: 10331.7 ± 2537.6, *p* = 0.018; MIAPaCa2-EPLIN^KD^-ravoxertinib: 18816.7 ± 3286.6, *p* > 0.05; Hour 3: MIAPaCa2-WT-ravoxertinib: 21373.7 ± 10452.8, *p* = 0.036; MIAPaCa2-EPLIN^KD^-ravoxertinib: 26563.7 ± 3841.9, *p* > 0.05; Hour 4: MIAPaCa2-WT-ravoxertinib: 28323.3 ± 11,950, *p* = 0.024; MIAPaCa2-EPLIN^KD^-ravoxertinib: 41965.3 ± 4081.5, *p* > 0.05; Representative pictures of Hour 0 and Hour 4 from each model are shown. (**E**) Wound Scratching based migration assays on PANC1 models along with wortmannin. Mean closed area (pixel): Hour 1: PANC1-WT: 15883.7 ± 3846, PANC1-WT-wortmannin: 4618.7 ± 2395.8, *p* = 0.013; PANC1-EPLIN^KD^: 6854.7 ± 5861.8, PANC1-EPLIN^KD^-wortmannin: 8072 ± 5657.2, *p* > 0.05; Hour 2: PANC1-WT: 47033.3 ± 15156.1, PANC1-WT-wortmannin: 12138.3 ± 1150.1, *p* = 0.017; PANC1-EPLIN^KD^: 20362.3 ± 4679.9, PANC1-EPLIN^KD^-wortmannin: 37,423 ± 4000.3, *p* = 0.009; Hour 3: PANC1-WT: 77,017 ± 18272.3, PANC1-WT-wortmannin: 30094.3 ± 9630.3, *p* = 0.017; PANC1-EPLIN^KD^: 33961.7 ± 11108.5, PANC1-EPLIN^KD^-wortmannin: 53141.7 ± 13481.6, *p* > 0.05; Hour 4: PANC1-WT: 94703.7 ± 19701.7, PANC1-WT-wortmannin: 35147.3 ± 10,317, *p* = 0.0097; PANC1-EPLIN^KD^: 52384.7 ± 8230.9, PANC1-EPLIN^KD^-wortmannin: 62301.7 ± 17,178, *p* > 0.05; (**F**) Wound Scratching based migration assays on PANC1 models along with LY294002. Mean closed area (pixel): Hour 1: PANC1-WT-LY294002: 1940.3 ± 1420.3, *p* = 0.0042; PANC1-EPLIN^KD^-LY294002: 7576.3 ± 4479.1, *p* > 0.05; Hour 2: PANC1-WT-LY294002: 18,047 ± 5557.9, *p* = 0.036; PANC1-EPLIN^KD^-LY294002: 25792.7 ± 12078.2, *p* > 0.05; Hour 3: PANC1-WT-LY294002: 26,579 ± 9415.59, *p* = 0.013; PANC1-EPLIN^KD^-LY294002: 42604.7 ± 19277.8, *p* > 0.05; Hour 4: PANC1-WT-LY294002: 31,929 ± 10929.4, *p* = 0.0085; PANC1-EPLIN^KD^-LY294002: 60,952 ± 25168.9, *p* > 0.05; (**G**) Wound Scratching based migration assays on PANC1 models along with PD98059. Mean closed area (pixel): Hour 1: PANC1-WT-PD98059: 794.5 ± 94, *p* = 0.013; PANC1-EPLIN^KD^-PD98059: 1370.7 ± 378.3, *p* > 0.05; Hour 2: PANC1-WT-PD98059: 20439.5 ± 7414, *p* > 0.05; PANC1-EPLIN^KD^-PD98059: 20,718 ± 8298.3, *p* > 0.05; Hour 3: PANC1-WT-PD98059: 32,521 ± 5137.8, *p* = 0.049; PANC1-EPLIN^KD^-PD98059: 34712.3 ± 10104.9, *p* > 0.05; Hour 4: PANC1-WT-PD98059: 42754.5 ± 3704.5, *p* = 0.039; PANC1-EPLIN^KD^-PD98059: 53546.3 ± 13910.5, *p* >  0.05. (**H**) Wound Scratching based migration assays on PANC1 models along with ravoxertinib. Mean closed area (pixel): Hour 1: PANC1-WT-ravoxertinib: 505.5 ± 840.7, *p* = 0.013; PANC1-EPLIN^KD^-PD98059: 6639 ± 5462.6, *p* > 0.05; Hour 2: PANC1-WT-ravoxertinib: 10430.5 ± 2180, *p* = 0.048; PANC1-EPLIN^KD^-PD98059: 30963.3 ± 6208.2, *p* > 0.05; Hour 3: PANC1-WT-ravoxertinib: 30,277 ± 6093.8, *p* = 0.044; PANC1-EPLIN^KD^-PD98059: 54194.7 ± 2700, *p* = 0.037; Hour 4: PANC1-WT-ravoxertinib: 47,554 ± 629.3, *p* = 0.049; PANC1-EPLIN^KD^-PD98059: 69335.7 ± 6059.7, *p* = 0.045. Representative pictures of Hour 0 and Hour 4 from each model are shown. *N* = 3, data shown represents mean ± SD. * represents *p* < 0.05, ** represents *p* < 0.001.

Enlightened by our analysis, we performed wound scratching assays on the in vitro models along with two PIK3 kinases inhibitors, Wortmannin and LY294002, as well as two MEK and ERK inhibitors, namely PD98059 and ravoxertinib (Fig. 8). As Fig. 8A indicated, cell migration rates were decreased significantly after knocking down EPLIN following by treating cells with Wortmannin compared to MIAPaCa2-WT group. Comparing to MIAPaCa2-EPLIN^KD^, there was no difference in migration rates noted when the knocked down model was supplemented with Wortmannin. Treating MIAPaCa2-WT cells with another PIK3 inhibitor, LY294002 also resulted in inhibition of cell migration significantly at hour 1, hour 3 and hour 4. While LY294002 did not inhibit migration in the MIAPaCa2-EPLIN^KD^ group (Fig. 8B). The two MIAPaCa2 models were also supplemented with a MEK inhibitor, PD98059, to perform wound scratching assays (Fig. 8C). PD98059 inhibited migration in MIAPaCa2-WT cells significantly at hour 1, 3 and 4. While it did not decrease migration rates in the EPLIN knocked down model. Addtionally, as Fig. 8D showed, cell migration rates were decreased significantly when MIAPaCa2-WT cells were supplemented with the ERK inhibitor, ravoxertinib. Such downregulation of cell migration rates was not noted when EPLIN was knocked down in MIAPaCa2 cells and was supplemented with ravoxertinib. Similar results were also observed in the PANC1 model (Fig. 7E, F, G & H). As Fig. 8E demonstrated, treating PANC1-WT cells with Wortmannin resulted in a significant slower migration. Meanwhile the EPLIN knocked down group treating with Wortmannin did not result in an inhibition of cell migration. In the PANC1-WT group, treating cells with LY294002 decreased cell migration significantly. Whlist treating PANC1-EPLIN^KD^ cells with it did not result in a change of cell migration (Fig. 8F). Similarly, PD98059 inhibited cell migration significantly in PANC1 cells at hour 1, 3 and 4. No change of cell migration was noted in the EPLIN knocked down PANC1 cells after treating with PD98059 (Fig. 8G). Furthermore, as Fig. 8H significant inhibition of cell migration was also noted when PANC1-WT cells was supplemented with ravoxertinib. Ravoxertinib did not inhibit cell migration when EPLIN was knocked down in PANC1 cells. Hence, we propose that the ability of the inhibitors described above in inhibiting cellular migration in pancreatic cancer, requires the presence of EPLIN. EPLIN may therefore regulate cell migration by mediating downstream signalling of PI3Ks and MAPK.

## Discussion

Over the past decade, EPLIN has been broadly studied and established as a tumour suppressor in a number of cancer types^13,20,21,23,24,33,34^. We previously reported the EPLIN expression profile in pancreatic cancer by comparing the ratio between normal and tumour tissues which can be misleading^35^. However, the results reported here by presenting levels of DRIM in tumour tissues strongly suggest that EPLIN may not be a suppressor in pancreatic cancer. In our current study, EPLIN demonstrated an oncogenic role. Firstly, unlike its expression profile in other cancer types, higher expression levels of EPLIN was observed in pancreatic cancer tissues compared to the normal tissues, at both mRNA and protein levels. Such upregulation also contributed to poorer survival outcomes of pancreatic cancer patients. These intriguing findings propose an inverse role for EPLIN to other cancer type, such as breast cancer^24^ and colorectal cancer^23^ and is of significant interest to researchers who are researching for novel therapeutic targets for pancreatic cancer.

Secondly, pancreatic cancer and its associated high mortality rates have made it a great threat to public health. Hence, understanding key mechanisms underlying development/progression and sensitivity to various treatments is of paramount importance to aid in the outcomes of patients affected by this cancer. EPLIN is an actin binding protein, which allows stabilisation of actin dynamics by virtue of it’s actin binding sites^14^. EPLIN also takes part in maintaining the structure of the cytoskeleton as well as adherens junctions, by interacting with the cadherin-catenin complex^15^. The downregulation or inactivation of EPLIN will lead to the disruption of actin dynamics and allow cancer cells to gain more potential to migrate and invade. Therefore, EPLIN was also shown to be a profound regulator for the EMT process^16,18,36^. Interestingly, by performing cellular growth and migration assays with EPLIN manipulated pancreatic cancer cell models, we observed reduced growth and migration rates after knocking down EPLIN. This suggests that the presence of EPLIN promotes these important cellular functions, which allows the tumour to grow and disseminate. The mechanism behind the suppressive role that EPLIN exhibits was already elucidated in previous studies^19,21,22^. In order to shed light on the investigation between EPLIN and its reported interacting partners and mechanism behind action in pancreatic cancer, we revealed that EPLIN regulated expression level of SNAIL, SLUG and ZEB1, three important contributors to the EMT process, positively. These findings showed the EPLIN may play a different role during EMT in pancreatic cancer than it does in other epithelial cancer types^16,18,36^. Hence, our new data demonstrates it will be necessary to hunt for further investigation to fully understand the relationship between EPLIN and its established interacting partners in pancreatic cancer, given the opposite role it has in this cancer type. Aguilar-Valdés *et al. (2023)*, reported the observation of EPLIN upregulation in pancreatic cancer cells, which were adaptive resistant to MEK and PI3K kinase targeted therapy^37^. Indeed, KRAS mutation occurs frequently in pancreatic cancer and accounts for over 90% of cases of pancreatic cancer^38^. After activation of KRAS, two parallel signalling pathways are often promoted in pancreatic cancer, namely MAPK and PIK3CA^39^. Researchers have been studying small molecules to inhibit such signalling events to improve patient prognosis^39^. After analysing the correlation between EPLIN and key components in these two parallel signalling events by carrying out analysis on the TCGA dataset and western blotting, we reported that the mRNA and protein levels of EPLIN correlate with key players in the PIK3CA and MAPK signalling pathways. Notably, EPLIN regulates the phosphorylation of ERK1/2 in pancreatic cancer cells. ERK1/2 was reported to be a key upstream regulator to phosphorylate EPLIN when it acts as a tumour suppressor^17^. Besides, EPLIN regulates two family members of the RTK family who are responsible for activating MAPK and PIK3CA-AKT signalling^39^. Following by the observation of the positive correlation between EPLIN and key players in such signalling, we proposed that EPLIN might act as an essential upstream regulator of MAPK and PIK3CA-AKT signalling. Hence, EPLIN could be a valuable potential target for deactivating these signallings in pancreatic cancer and contribute to a better prognosis. We also demonstrated that the inhibition of EPLIN led to a failure in the inhibitory ability of Wortmannin, LY294002, PD98059 and ravoxertinib on cellular migration. Wortmannin^40^ and LY294002^41^ are specific selective PIK3 kinases inhibitors and have been reported to inhibit cellular migration in pancreatic cancer^42,43^. So did the ERK1/2 inhibitor, ravoxertinib^44,45^ and the MEK inhibitor, PD98059^46^. These findings in the current study emphasized the possibility that EPLIN acts as an upstream regulator of one of the signalling events that PIK3 kinases is involved in, as well as MAPK. It also highlights the potential that EPLIIN has to be developed as novel target for treating pancreatic cancer. Our findings are in line with the study Valdés *et al. (2023)* reported^37^, which highlights the relationship EPLIN has with MAPK and PIK3 signalling events in pancreatic cancer. As we discussed above, one of the key functions EPLIN has is in regulating cellular migration. A recent study reported that p62, a key regulator of autophagy, regulates and interacts with EPLIN to enhance cellular migration in oesophageal squamous cell carcinoma^47^. Such findings might suggest several novel signalling pathways involved in the mechanism behind EPLIN’s oncogenic role in pancreatic cancer.

Thirdly, pancreatic cancer is one of the most fatal cancer types due to difficult and late diagnosis. Poor survival rates and drug resistance have made it a serious burden to public health. Small molecules such as EGFR, HER2^48^ and PI3K^49^ were studied extensively as potential targets for treating pancreatic cancer. In this study, we demonstrated that inhibition of EPLIN led to a more sensitive response to gemcitabine and 5FU in pancreatic cancer cells. Furthermore, a similar effect was also seen with neratinib in PANC1 models. We reported that EPLIN was involved in regulating drug resistance in gastric cancer^50^ and colorectal cancer^23^. Combined with the current study, these findings implied the potential of targeting EPLIN to more sensitive chemotherapies and EGFR/HER2 targeted therapeutic response. In the past year, we have also reported that EPLIN regulates the expression levels of EGFR family members in colorectal cancer^23^. Lately, we observed a more sensitive response to neratinib when EPLIN was inhibited. In the current study, we also reported a positive correlation between EPLIN and EGFR, HER2 and HER3 in the TCGA cohort. Inhibition of EPLIN was also related to downregulation of some of family members based on the in vitro work. It would be useful to investigate if EGFR family members are involved in the interacting network of EPLIN in pancreatic cancer, given that the family is also crucial for activating MAPK and PIK3 kinases related signalling events. This area of research is still ongoing in the host lab.

EPLIN has two isoforms, previous studies of EPLIN mainly focused on the α isoform^12,23–25^, while the β isoform has gained more and more interest in recent years. The β isoform was reported to be involved in regulating actin dynamics in endothelial cells^51^. Of note, Li *et al. (2023)*, reported that EPLINβ worsened patient survival and promoted cellular migration in colorectal cancer^52^. Indeed, unlike downregulation of EPLINα which was frequently observed, EPLINβ was reported to be upregulated or unchanged in a previous study^34^. In the current study, the inhibition of EPLIN was performed by knocking down both isoforms. Our findings indicate either one or two isoforms promote pancreatic cancer progression. It would be interesting to explore the role the two isoforms play in pancreatic cancer, together with the prospect of their contribution to the oncogenic role played by EPLIN in pancreatic cancer.

## Electronic supplementary material

Below is the link to the electronic supplementary material.

Supplementary Material 1

## Data Availability

Data presented in this publication can be obtained from the lead author upon reasonable request.
