# Supplementary material for: EPLIN, a prospective oncogenic molecule with contribution to growth, migration and drug resistance in pancreatic cancer
Source: Sci Rep. 2024 Dec 28;14:30850. doi: 10.1038/s41598-024-81485-w (PMC11680852; doi:10.1038/s41598-024-81485-w)
Supplement: Supplementary file 1 — Supplementary Material 1 [file 41598_2024_81485_MOESM1_ESM.pdf]

S1

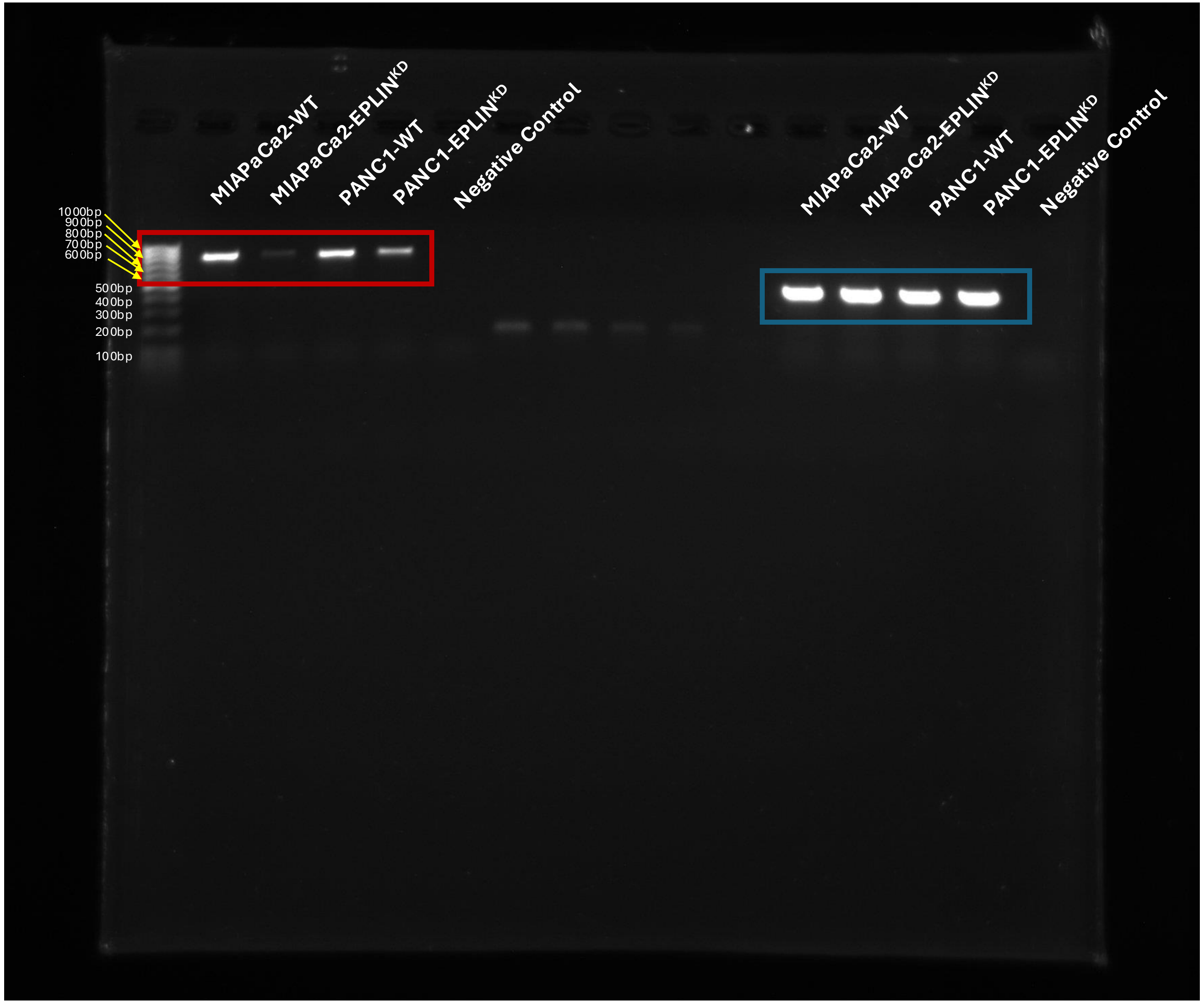

Figure S1. Uncropped gel electrophoresis result from conventional PCR probing EPLIN. Primers of EPLIN were used to probe EPLIN in the pancreatic cancer cell models (shown on the left), red box indicates the result of EPLIN demonstrated in Figure4A. Primers of GAPDH were utilised to probe GAPDH in the pancreatic cancer cell models (shown on the right). Blue box indicates the result of GAPDH shown in Figure 4A. Predicated product sizes: GAPDH:470 bp; EPLIN: 878 bp.

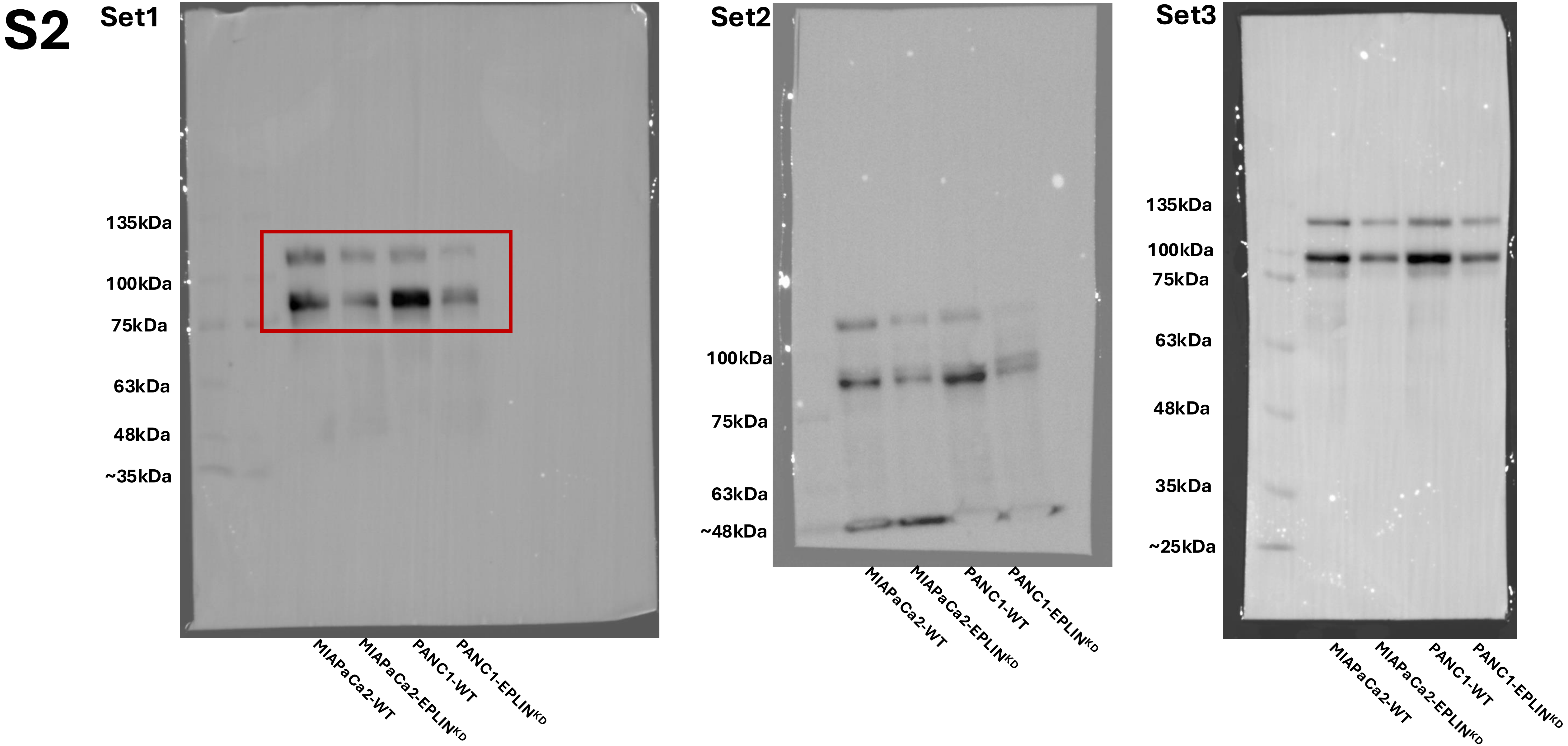

Figure S2. Uncropped western blotting of probing EPLIN. Set1, set2 and set3 are the independent replicated results of probing EPLIN in the pancreatic cancer cell models. Red box indicates the WB results probing EPLIN shown in Figure 4C. Ladders was labelled in each set. Predicated molecular weight of EPLIN: EPLINα: 90kDa, EPLINβ: 110kDa.

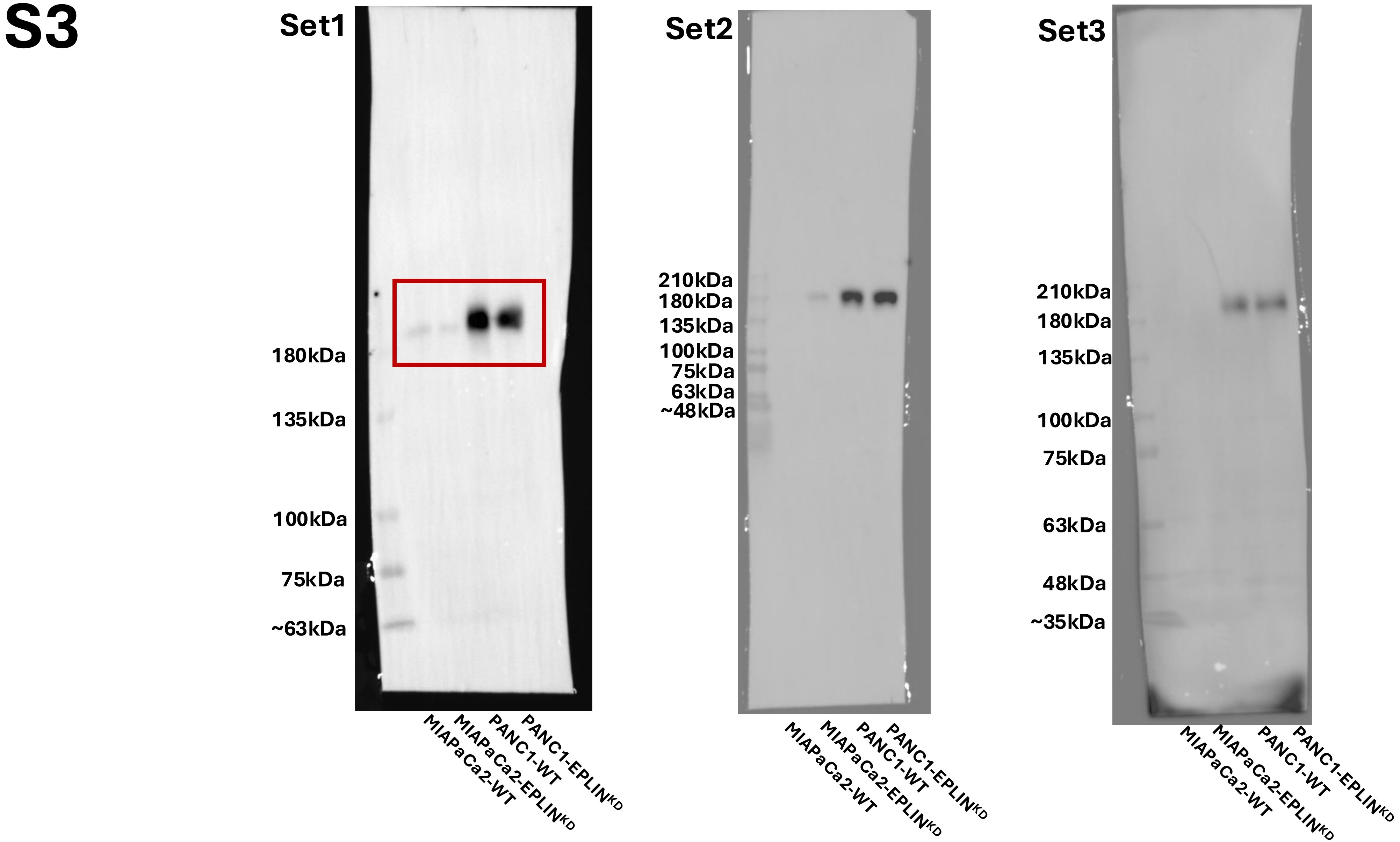

Figure S3. Uncropped western blotting of probing EGFR. Set1, set2 and set3 are the independent replicated results of probing EGFR in the pancreatic cancer cell models. Red box indicates the WB results probing EGFR shown in Figure 7B. Ladders was labelled in each set. Predicated molecular weight of EGFR: 170kDa.

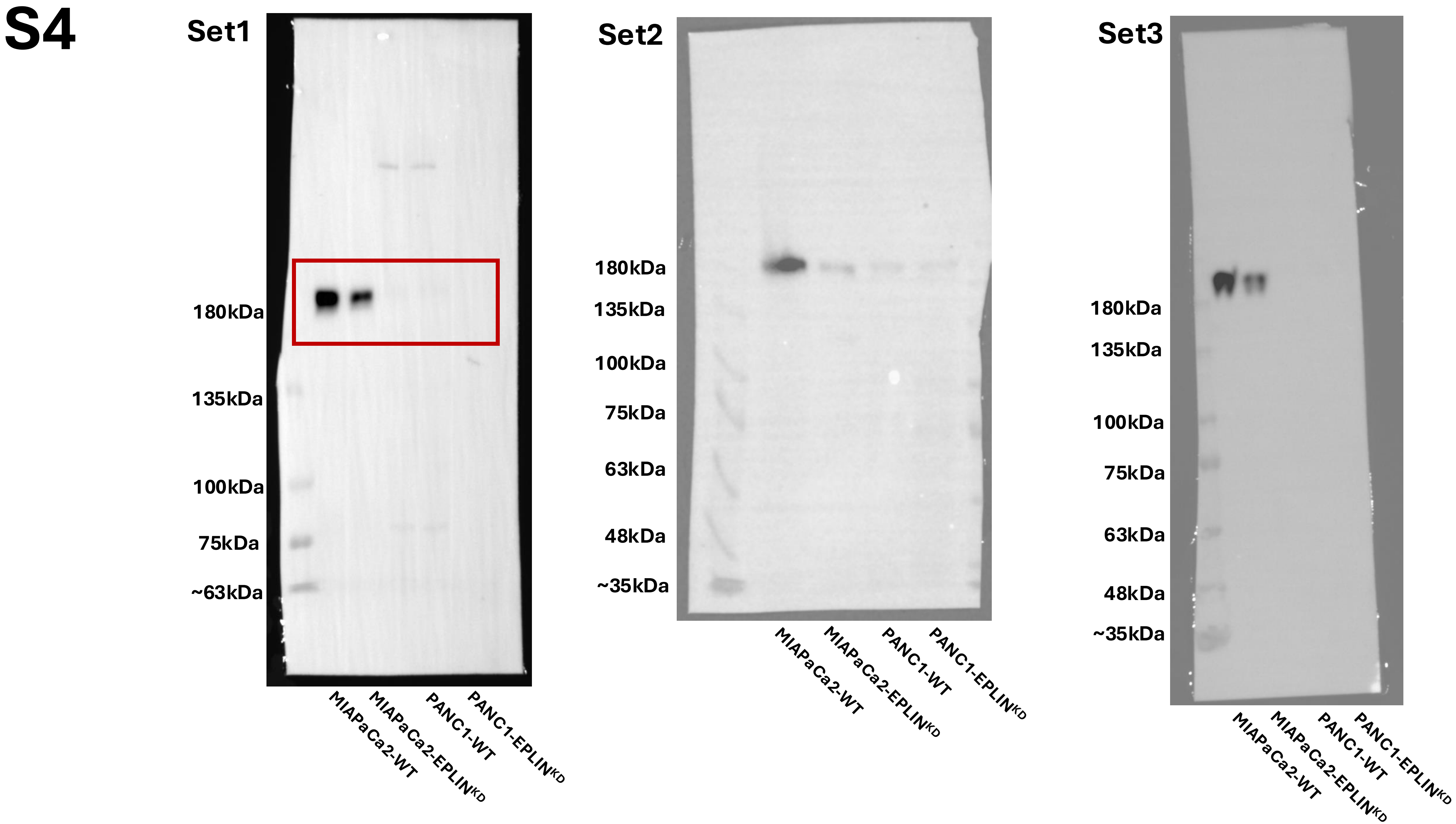

Figure S4. Uncropped western blotting for probing HER2. Set1, set2 and set3 are the independent replicated results of probing HER2 in the pancreatic cancer cell models. Red box indicates the WB results probing HER2 shown in Figure 7B. Ladders was labelled in each set. Predicated molecular weight of HER2: 185kDa.

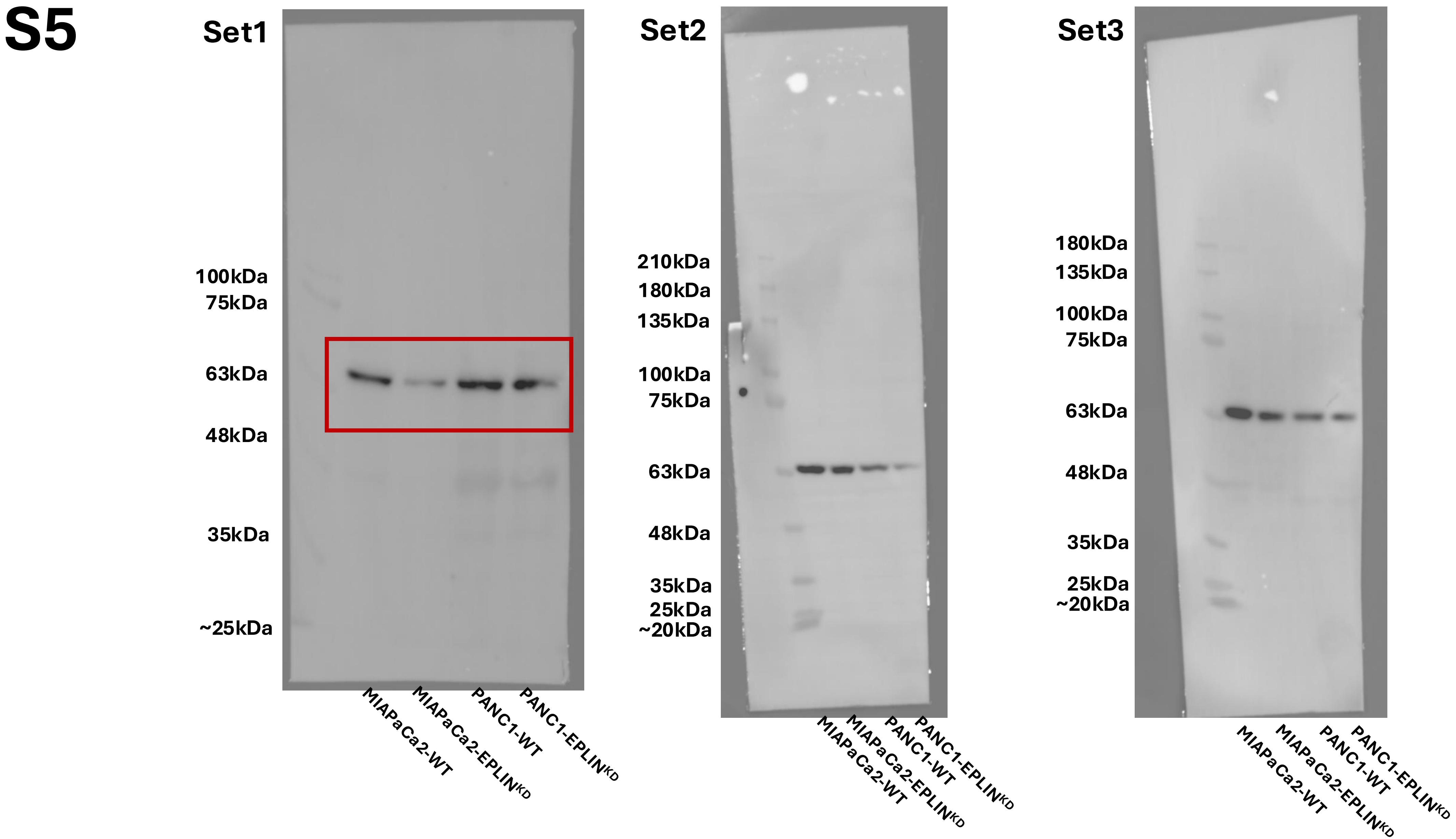

Figure S5. Uncropped western blotting for probing AKT1. Set1, set2 and set3 are the independent replicated results of probing AKT1 in the pancreatic cancer cell models. Red box indicates the WB results probing AKT1 shown in Figure 7B. Ladders was labelled in each set. Predicated molecular weight of AKT1: 62kDa.

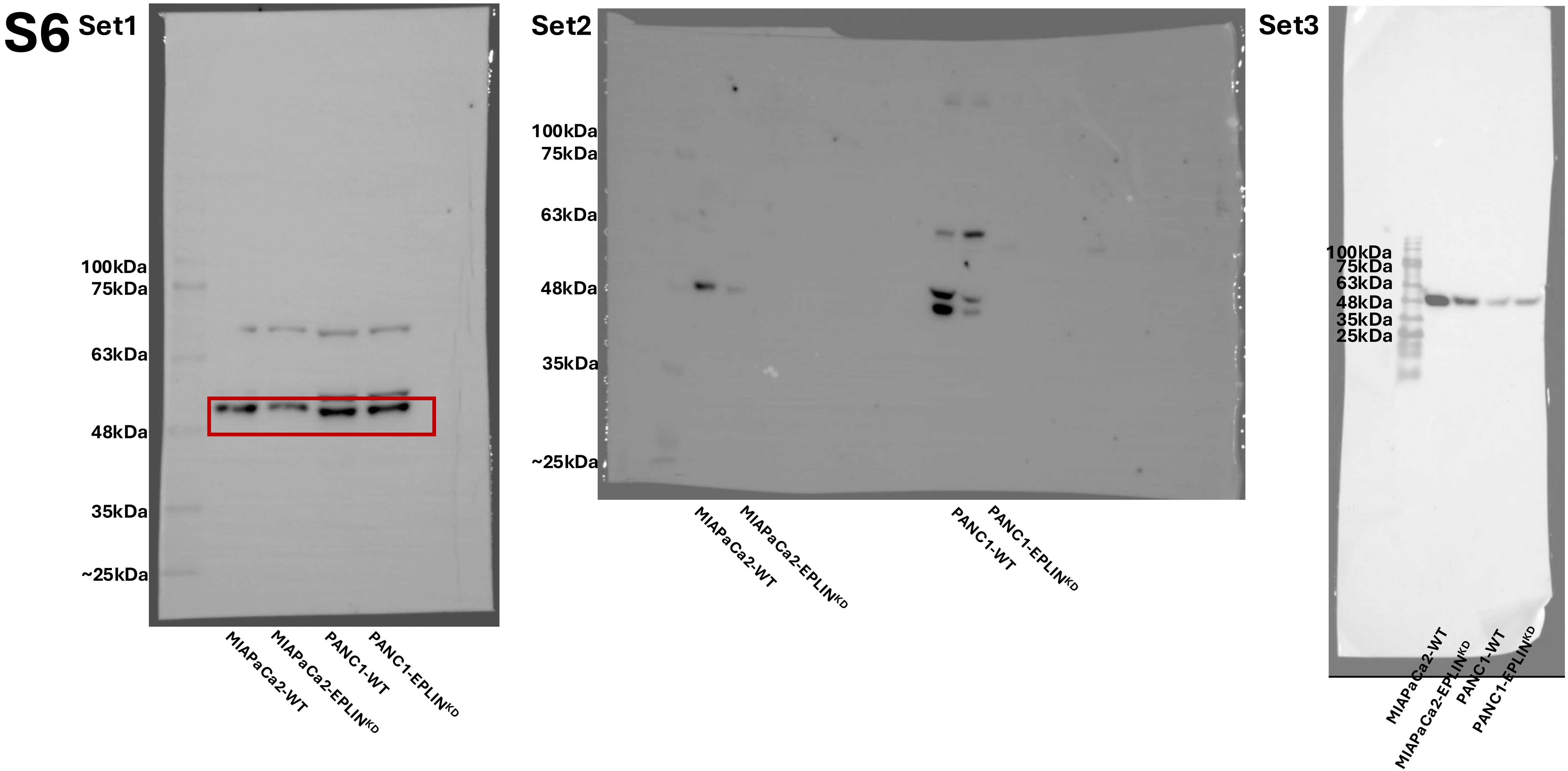

Figure S6. Uncropped western blotting for probing MEK2. Set1, set2 and set3 are the independent replicated results of probing MEK2 in the pancreatic cancer cell models. Red box indicates the WB results probing MEK2 shown in Figure 7B. Ladders was labelled in each set. Predicated molecular weight of MEK2: 47kDa.

S7

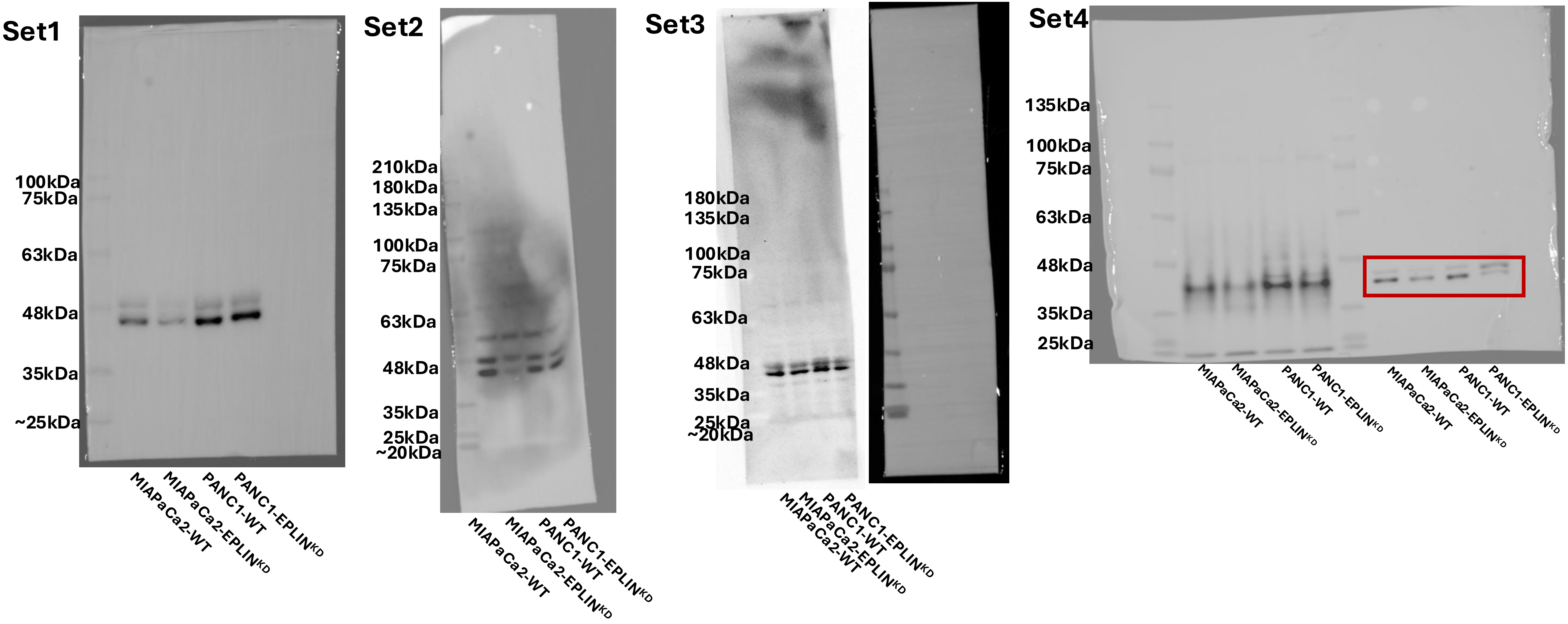

Figure S7. Uncropped western blotting for probing ERK1/2. Set1, set2, set3 and set4 are the independent replicated results of probing ERK1/2 in the pancreatic cancer cell models. Red box indicates the WB results probing ERK1/2 shown in Figure 7B. Ladders was labelled in each set. Predicated molecular weight of ERK1/2: ERK1: 44kDa, ERK2: 42kDa.

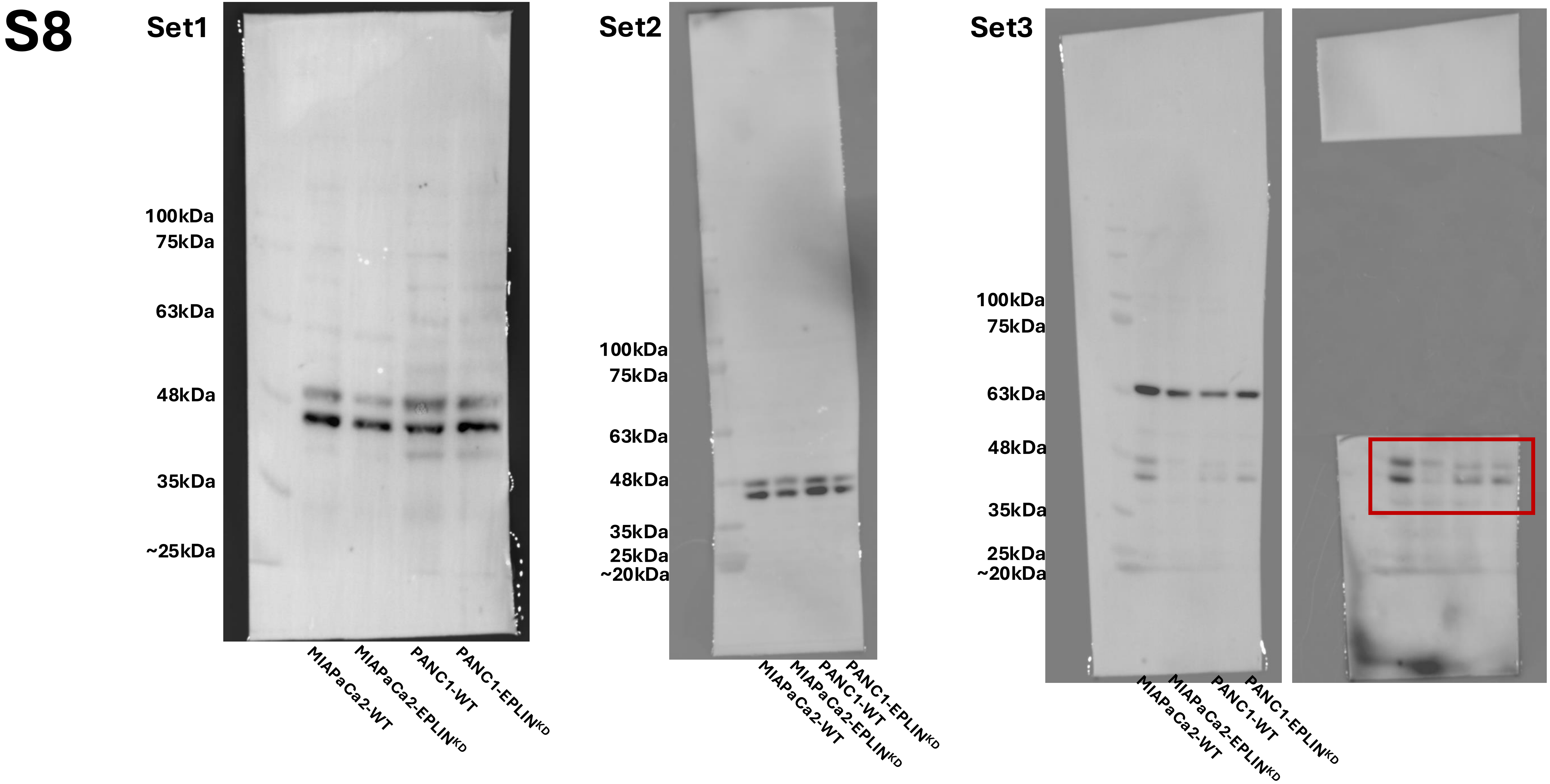

Figure S8. Uncropped western blotting for probing pERK1/2. Set1, set2 and set3 are the independent replicated results of probing pERK1/2 in the pancreatic cancer cell models. Red box indicates the WB results probing pERK1/2 shown in Figure 7B. Ladders was labelled in each set. Predicated molecular weight of pERK1/2: pERK1: 44kDa, pERK2: 42kDa.

S9

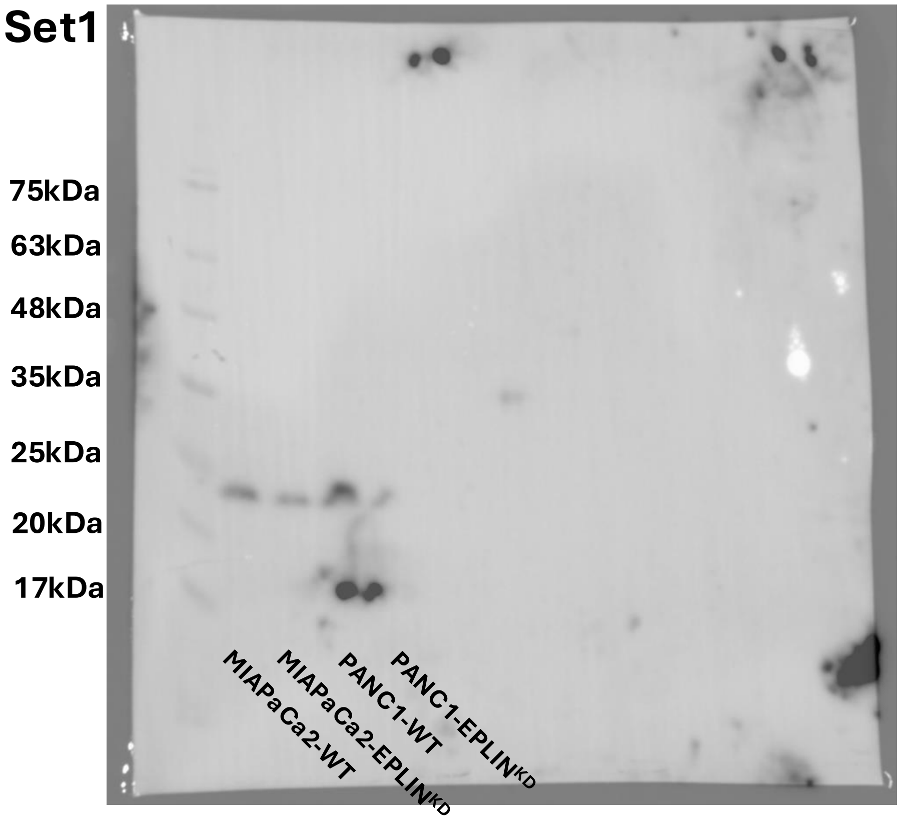

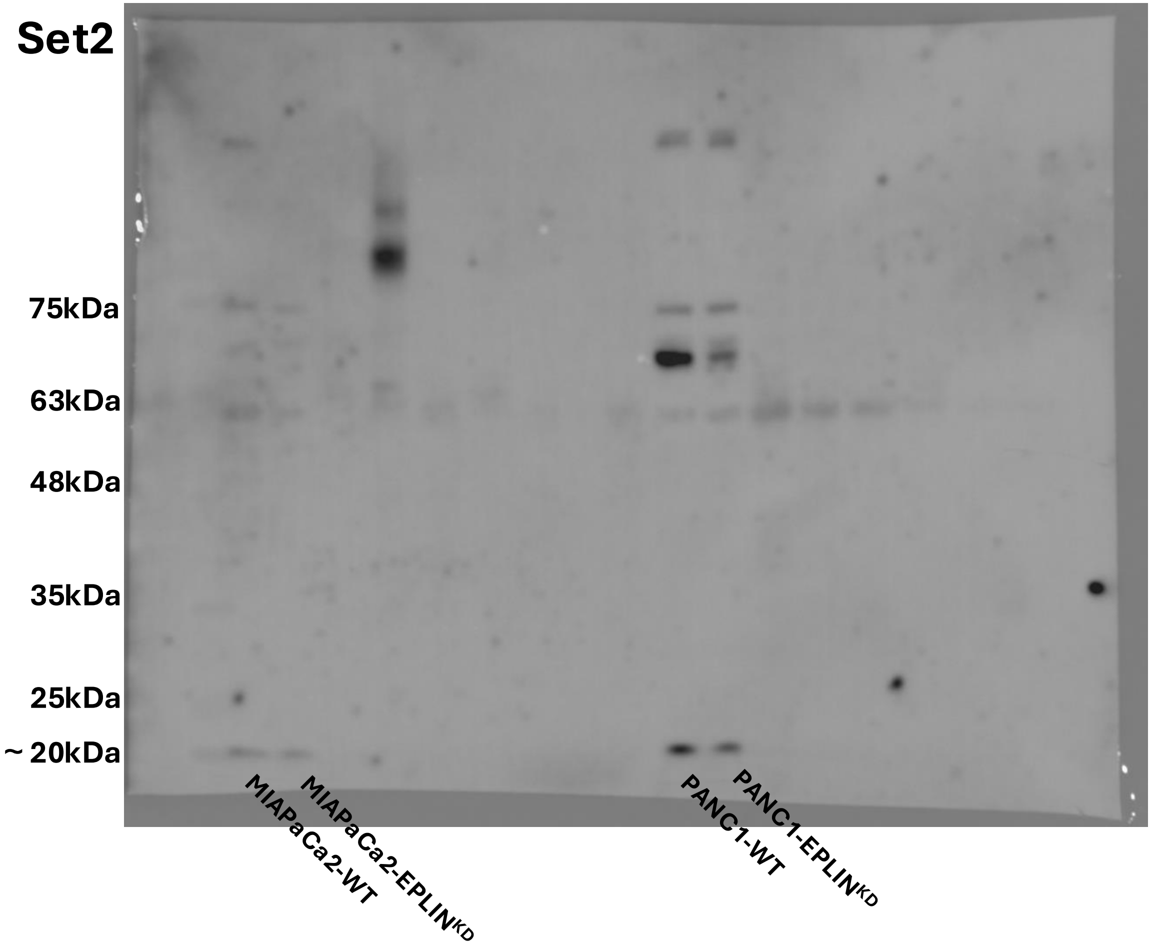

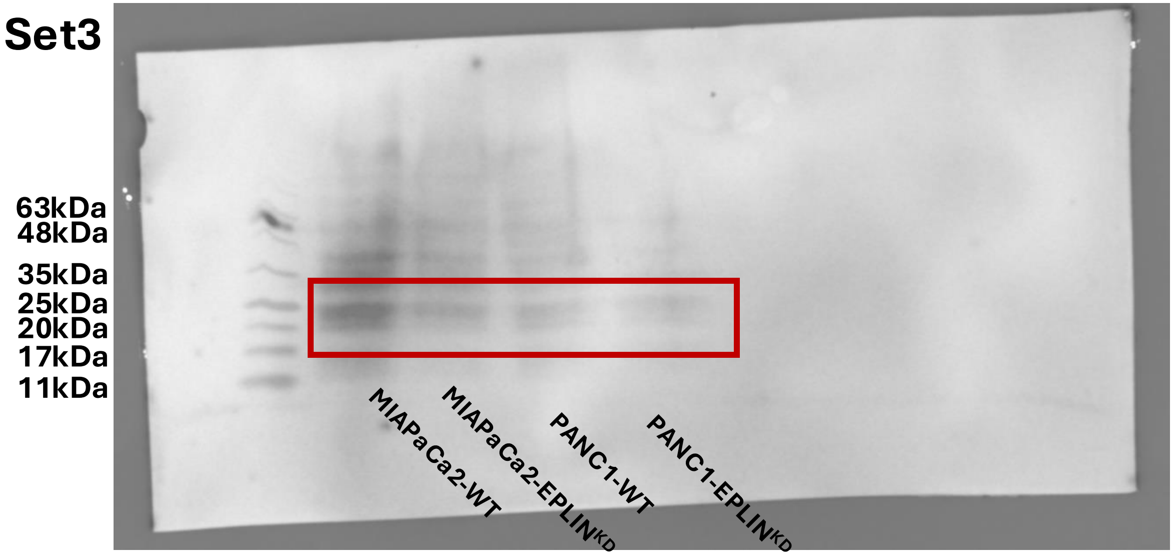

Figure S9. Uncropped western blotting for probing KRAS. Set1, set2 and set3 are the independent replicated results of probing KRAS in the pancreatic cancer cell models. Red box indicates the WB results probing KRAS shown in Figure 7B. Ladders was labelled in each set. Predicated molecular weight of KRAS: 21kDa.

S10

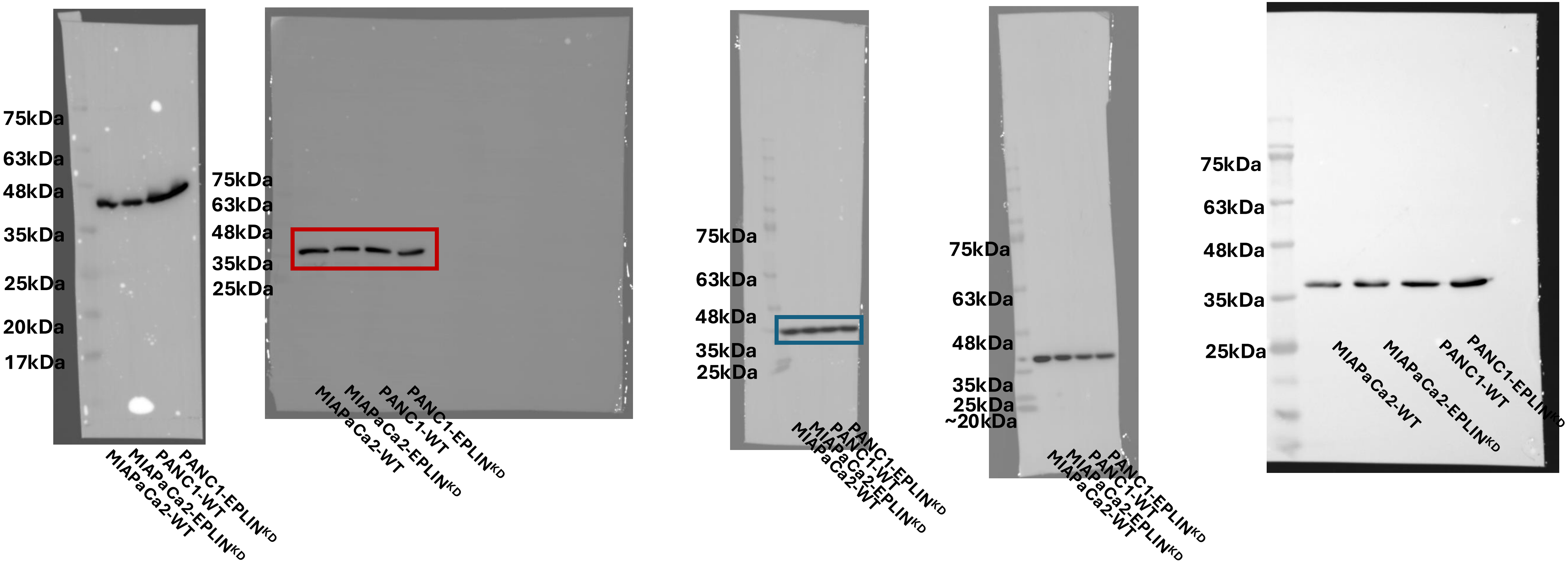

Figure S10. Uncropped western blotting for probing GAPDH. 5 independent replicated results of probing GAPDH in the pancreatic cancer cell models were demonstrated. Red box indicates the WB results probing GAPDH shown in Figure 4C. Blue box indicates the WB results probing GAPDH shown in Figure 7B. Ladders was labelled in each set. Predicated molecular weight of GAPDH: 37kDa.
